# Perioperative fluid management for adult cardiac surgery: network meta-analysis pooling on twenty randomised controlled trials

**DOI:** 10.1186/s13741-024-00440-5

**Published:** 2024-07-20

**Authors:** Yu-Tong Ma, Chen-Yang Xian-Yu, Yun-Xiang Yu, Chao Zhang

**Affiliations:** 1 Center for Evidence-Based Medicine and Clinical Research, Taihe Hospital, Hubei University of Medicine, No.32, Renmin South Road, Shiyan, 442000 Hubei China; 2 Department of Surgery, Taihe Hospital, Hubei University of Medicine, No.32, South Renmin Road, Shiyan, 442000 Hubei China

**Keywords:** Cardiac surgery, Perioperative period, Fluid management, Colloids, Crystalloids

## Abstract

**Background:**

The aim of this study was to evaluate colloids and crystalloids used in perioperative fluid therapy for cardiac surgery patients to further investigate the optimal management strategies of different solutions.

**Method:**

RCTs about adult surgical patients allocated to receive perioperative fluid therapy for electronic databases, including Ovid MEDLINE, EMBase, and Cochrane Central Register of Controlled Trials, were searched up to February 15, 2023.

**Results:**

None of the results based on network comparisons, including mortality, transfuse PLA, postoperative chest tube output over the first 24 h following surgery, and length of hospital stay, were statistically significant. Due to the small number of included studies, the results, including acute kidney injury, serum creatinine, serum microglobulin, and blood urea nitrogen, are from the direct comparison. For transfusion of RBCs, significant differences were observed in the comparisons of 3% gelatine vs. 6% HES 200/0.5, 4% albumin vs. 5% albumin, 4% gelatine vs. 5% albumin, 5% albumin vs. 6% HES 200/0.5, and 6% HES 130/0.4 vs. 6% HES 200/0.5. In transfusion of FFP, significant differences were observed in comparisons of 3% gelatine vs. 4% gelatine, 3% gelatine vs. 6% HES 200/0.5, 5% albumin vs. 6% HES 200/0.5, 4% gelatine vs. 5% albumin, 4% gelatine vs. 6% HES 200/0.4, and 6% HES 130/0.4 vs. 6% HES 200/0.5. For urinary output at 24 h after surgery, the results are deposited in the main text.

**Conclusion:**

This study showed that 3% gelatin and 5% albumin can reduce the transfuse RBC and FFP. In addition, the use of hypertonic saline solution can increase urine output, and 5% albumin and 6% HES can shorten the length of ICU stay. However, none of the perioperative fluids showed an objective advantage in various outcomes, including mortality, transfuse PLA, postoperative chest tube output over the first 24 h following surgery, and length of hospital stay. The reliable and sufficient evidences on the injury of the kidney, including acute kidney injury, serum creatinine, serum microglobulin, and blood urea nitrogen, was still lacking. In general, perioperative fluids had advantages and disadvantages, and there were no evidences to support the recommendation of the optimal perioperative fluid for cardiac surgery.

**Supplementary Information:**

The online version contains supplementary material available at 10.1186/s13741-024-00440-5.

## Introduction

Patients undergoing cardiac surgery usually require haemodynamic support immediately following surgery, and fluid therapy is currently the primary approach to maintain perioperative haemodynamic stability (Heming et al. 2020), which has a significant impact on the prognosis of these patients (Srinivasa and Hill 2012). During the perioperative period, fluid management is used to maintain the perfusion of the patients’ vital organs and to avoid hypovolaemia, inadequate tissue perfusion, and tissue oedema and cardiovascular complications caused by the infusion of large amounts of blood and blood products (Cooke and Snyder 1998; Patil and Salunke 2020). A restriction was putting forward in 2013 by the European Medical Association that they restricted HES application which mandated changes in common use volume regimes and caused the shift from colloids to crystalloids. One retrospective single-center study compares the volume management before and after this EMA decision (Datzmann et al. 2022). A large number of safety studies confirming the adverse effects of HES in patients with acute kidney injury and bleeding coagulation have been based mainly on patients with sepsis and critical illness, which is one of the main reasons why HES was banned by the European Medicines Agency. However, the nephrotoxicity of HES in cardiac surgery patients remains a topic of debate, and no previous study has attempted a comprehensive comparative evaluation of the safety of HES and a variety of fluid therapies used in cardiac surgery patients. Moreover, studies on the safety of albumin use in cardiac surgery patients have yielded conflicting findings (Matebele et al. 2020), and the safety of different fluid therapies in cardiac surgery mainly in terms of postoperative blood transfusion, renal function, and mortality, remains uncertain. The existing studies on the effects of fluid management in adult cardiac surgery are traditional meta-analyses that do not rank the current clinical benefits of all relevant fluid replacement options in terms of outcomes, limiting the extrapolation of clinical evidence. Therefore, the current study comprehensively evaluates the clinical efficacy of all types of colloids and crystalloids in order to facilitate perioperative fluid management reconstruction in patients undergoing cardiac surgery and provide evidence support for clinical decision-making.

## Methods

This network meta-analysis was developed in accordance with the Preferred Reporting Items for Systematic Reviews and Meta-analyses for Network Meta-Analyses (PRISMA-NMA) guidelines (Hutton et al. 2015).

### Literature search

The studies were selected among papers published in Ovid MEDLINE, EMBase, and Cochrane Central Register of Controlled Trials before February 15, 2023. Detailed electronic search strategies are presented in Supplementary Method 1.

### Inclusion and exclusion criteria

The inclusion criteria were as follows: (1) population, patients who underwent cardiac surgery; (2) interventions and control, 3% or 4% gelatine, 4% or 5% albumin, 6% HES, 6% HES 130/0.4 or 200/0.5, hyperosmolar sodium lactate, hypertonic saline solution, plasma protein fraction, or Ringer’s solution; (3) outcomes, mortality, number of patients who required transfusion of red blood cells (RBCs), number of patients who required transfusion of fresh frozen plasma (FFP), number of patients who required transfusion of platelets (PLA), acute kidney injury (AKI), serum creatinine, serum microglobulin, blood urea nitrogen, urinary output at 24 h after surgery, ostoperative chest tube output over the first 24 h following surgery, length of ICU stay, and length of hospital stay; (4) study design, randomised controlled trials (RCTs).

The criteria for exclusion were as follows: (1) repeated studies, (2) studies with missing data, (3) studies from Boldt’s academic HES data, and (4) retracted studies.

### Data collection and processing

Two reviewers independently screened the literature selected according to the criteria and extracted relevant data on year, interventions, study design, and results. Differences were resolved by discussion, while disagreements were resolved by a third reviewer.

### Quality assessment

Two investigators independently evaluated all included studies using the Cochrane risk of bias tool for RCTs. The risk of bias was assessed in terms of the following five aspects: (1) bias arising from the randomisation process, (2) bias due to deviations from the intended intervention, (3) bias due to missing outcome data, (4) bias in measurement of the outcome, and (5) bias in the selection of the reported result.

### Statistical analysis

Dichotomous data were expressed as relative risk (RR) with the 95% confidence interval (CI), and continuous data were expressed as the mean difference (MD) or standardised mean differences (SMD) with the 95% CI. Heterogeneity between studies was assessed using the chi-square test, in which significance was set at *P* ≤ 0.1, and the *I*^2^ statistic. *I*^2^ ≥ 40% denoted significant heterogeneity, and a random-effects model was used. The fixed-effects model was used when *I*^2^ was < 40%. A network meta-analysis can provide reliable evidence for comparison of direct and indirect multiple interventions. A design-by-treatment interaction model designed by processing was adopted for network element analysis. The inconsistency between direct evidence and indirect evidence showed that the transmission between results was not obvious by the node-splitting method. To summarise the probability, we used the surface under the cumulative ranking curve (SUCRA) to provide a summary of the cumulative ranking. By definition, SUCRA values reflect the efficacy or safety of an intervention, and thus, the rank-heat plot with larger SUCRA scores implies more effective or safer interventions (Veroniki et al. 2016). All statistical analyses were performed using STATA 15.0. software, and it had obtained a copyright licence.

## Results

### Literature identification

**Fig. 1:**
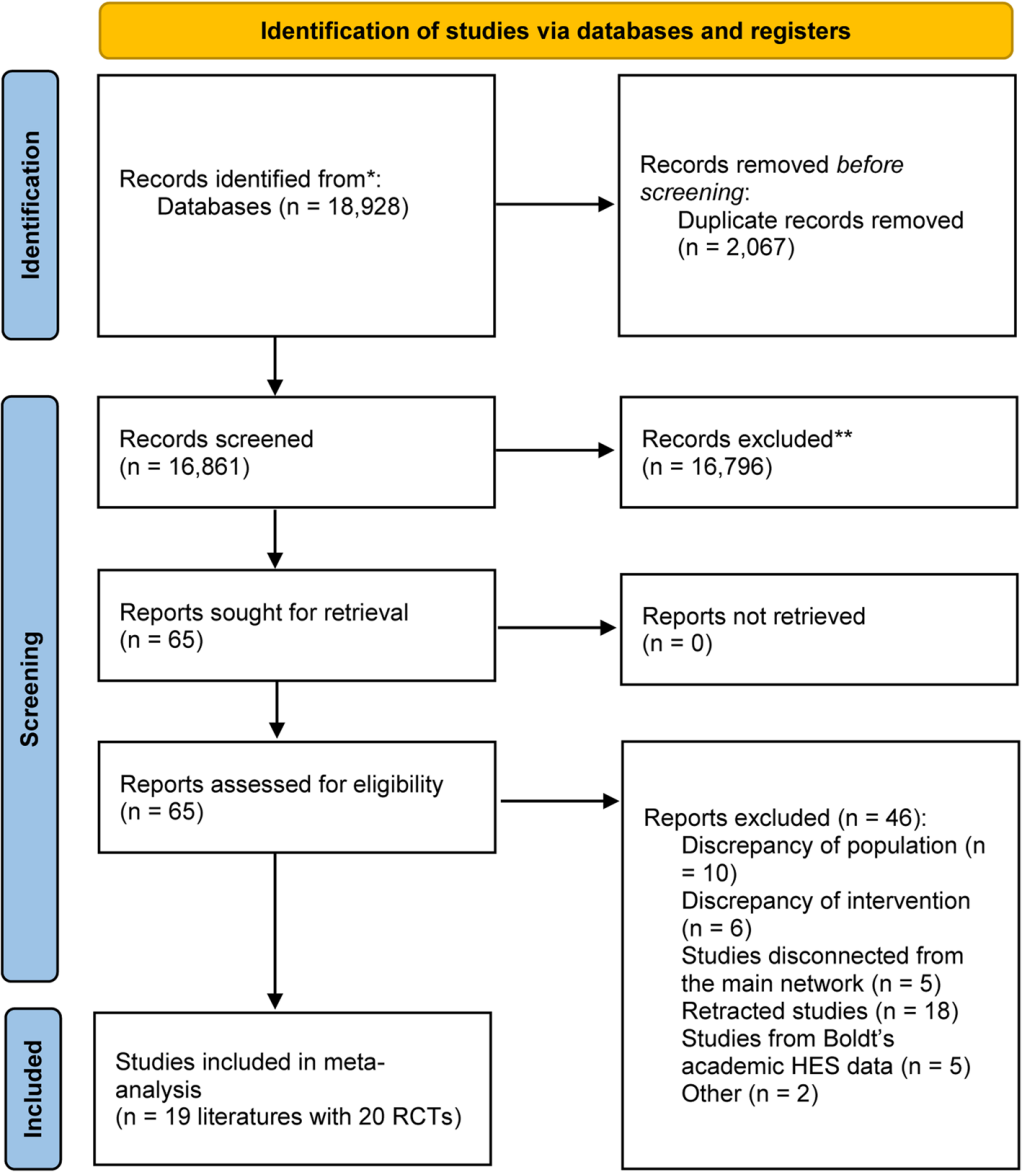
Study selection

Our literature search identified 18,928 records. After excluding 2067 duplicates and 16,861 records by screening the titles, 29 studies were included in the systematic review, as shown in Fig. 1. A total of 19 articles (Lee et al. 2021; Duncan et al. 2020; Öztürk et al. 2015; Skhirtladze et al. 2014; Boom et al. 2013; Alavi et al. 2012; Schramko et al. 2010a; Schramko et al. 2009; Niemi et al. 2008; Kuitunen et al. 2007; Niemi et al. 2006; Linden et al. 2005; Kasper et al. 2003; Gallandat Huet et al. 2000; Mazhar et al. 1998; Munsch et al. 1988; Belcher and Lennox 1984; Diehl et al. 1982; Schramko et al. 2010b) reporting 20 RCTs with a total number of 1497 participants were included in the network meta-analysis.

### Study characteristics and quality assessment

A total of 20 RCTs were included in this network meta-analysis, and the basic characteristics of each study, including patient age, sex, type of surgery, type of fluid infusion, and dose of fluid infusion are shown in Table 1. In addition, the risk of bias of the included studies was assessed, and the results are presented in Supplementary Table 1.

**Table 1:** Basic characteristics for included studies of network meta-analysis

Study	Year	Sample	Age (year)	Gender (M/F)	Type of cardiac surgery	Type 1 of perioperative fluid therapy	Type 2 of perioperative fluid therapy	Type 3 of perioperative fluid therapy
Alavi	2012	29; 31; 32	59 (11); 60 (8.7); 57 (10.4)	NR	On-pump coronary artery bypass	Ringer’s solution	4% gelatin	6% HES
Belcher	1983	30; 43	53 (35–70); 52 (42–62)	39/4; 29/1	Coronary artery disease	HES	Plasma protein fraction	NR
Boom	2013	48; 50	56.00 ± 6.57; 56.49 ± 8.42	NR	Coronary artery bypass	6% HES	Hyperosmolar sodium lactate	NR
Diehl	1982	33; 27	58.0 ± 8.0; 56.6 ± 8.1	29/4; 20/7	Coronary artery bypass	6% HES	4% HA	NR
Duncan	2020	69; 72	71 (10); 69 (9)	47/22; 44/28	Elective aortic valve replacement	6% HES 130/0.4	5% HA	NR
Huet	2000	30; 29	63.5 ± 9.0; 61.0 ± 10.3	25/5; 24/5	Coronary artery bypass grafting	6% HES 130/0.4	6% HES 200/0.5	NR
Kasper	2003	59; 58	63 ± 8; 64 ± 7	47/12; 46/12	Elective coronary artery bypass surgery	6% HES 130/0.4	6% HES 200/0.5	NR
Kuitunen	2007	15; 15; 15	60 (21–72); 67 (58–83); 61 (39–74)	12/3; 12/3; 12/3	Coronary artery bypass grafting	6% HES	4% succinylated gelatin	4% HA
Lee	2021	66; 69	71 ± 9.6; 69 ± 8.3	45/21; 41/28	Elective aortic valve replacement surgery	6% HES 130/0.4	5% HA	NR
Linden	2005	65; 68	67 ± 11; 66 ± 8	50/15; 48/20	Coronary surgery	6% HES 130/0.4	3% modified fluid gelatin	NR
Mazhar	1997	10; 10	57.1 ± 7.8; 60.1 ± 7.8	10/0; 7/3	Coronary artery bypass grafting	7.2% Hypertonic saline solution	Gelatin	NR
Munsch	1988	20; 20	59 (43–73); 55 (42–70)	17/3; 17/3	Elective coronary artery bypass surgery	6% HES	Plasma protein fraction	NR
Niemi	2008	15; 15; 15	59 (34–73); 63 (33–77); 61 (31–78)	11/4; 11/4; 9/6	On-pump cardiac surgery	6% HES 130/0.4	6% HES 200/0.5	4% HA
Niemi	2006	15; 15; 15	62 (21–72); 63 (39–74); 66 (58–83)	12/3; 12/3; 12/3	Cardiac surgery	6% HES 200/0.5	4% HA	4% succinylated gelatin
Öztürk a	2014	10; 10	62 ± 11; 59 ± 8	8/2; 7/3	NR	6% HES 130/0.4	4% modified gelatin solution	NR
Öztürk b	2014	24; 24	64.3 ± 13.2; 60.1 ± 8.9	18/6; 20/4	Coronary artery bypass surgery	6% HES 200/0.5	4% modified gelatin solution	NR
Schramko	2009	15; 15; 15	59 (39); 63 (44); 61 (48)	11/4; 11/4; 9/6	Cardiac surgery	6% HES 130/0.4	6% HES 200/0.5	4% HA
Schramko	2010	15; 15; 15	65 (46–84); 63 (50–78); 65 (50–77)	10/5; 9/6; 10/5	Elective primary cardiac surgery	6% HES 130/0.4	4% gelatin	Ringer’s acetate solution
Schramko	2010	15; 15; 15	65 (46–84); 63 (50–78); 65 (50–77)	10/5; 9/6; 10/5	Coronary artery bypass grafting	6% HES 130/0.4	4% gelatin	Ringer’s acetate solutions
Skhirtladze	2013	81; 76; 79	67 (28–87); 66 (23–85); 67 (24–87)	52/29; 53/23; 61/18	Elective cardiovascular surgery	6% HES 130/0.4	5% HA	Ringer’s lactate

Note: *HA* human albumin, *HES* hydroxyethyl starch, *NR* not reported, *M* male, *F* female

### Results of network and direct *meta*-analyses

#### Mortality

**Fig. 2:**
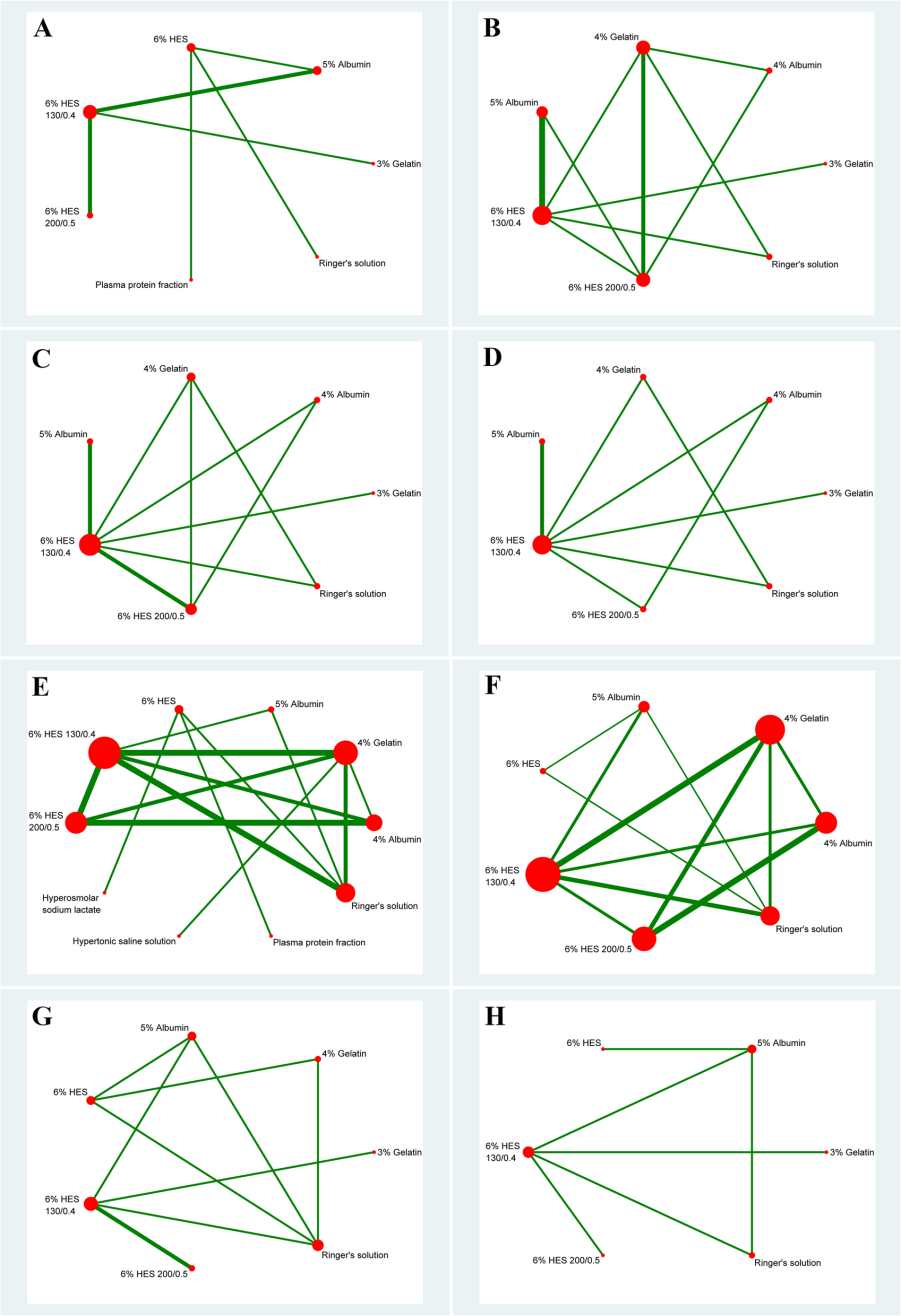
Network plot for all outcomes. Note: Mortality (**A**), transfuse red blood cell (**B**), transfuse fresh frozen plasma (**C**), transfuse platelet (**D**), urinary output at 24 h after surgery (**E**), postoperative chest tube output over the first 24 h following surgery (**F**), length of ICU stay (**G**), and length of hospital stay (**H**). The size of the nodes corresponds to the number of trials under study. The larger the node, the larger the number of participants in the study. The results of direct comparisons are connected by a line, the thickness of which corresponds to the sum of the sample sizes compared for each pairwise treatment. The thicker the line, the larger the sample size for comparison

**Fig. 3:**
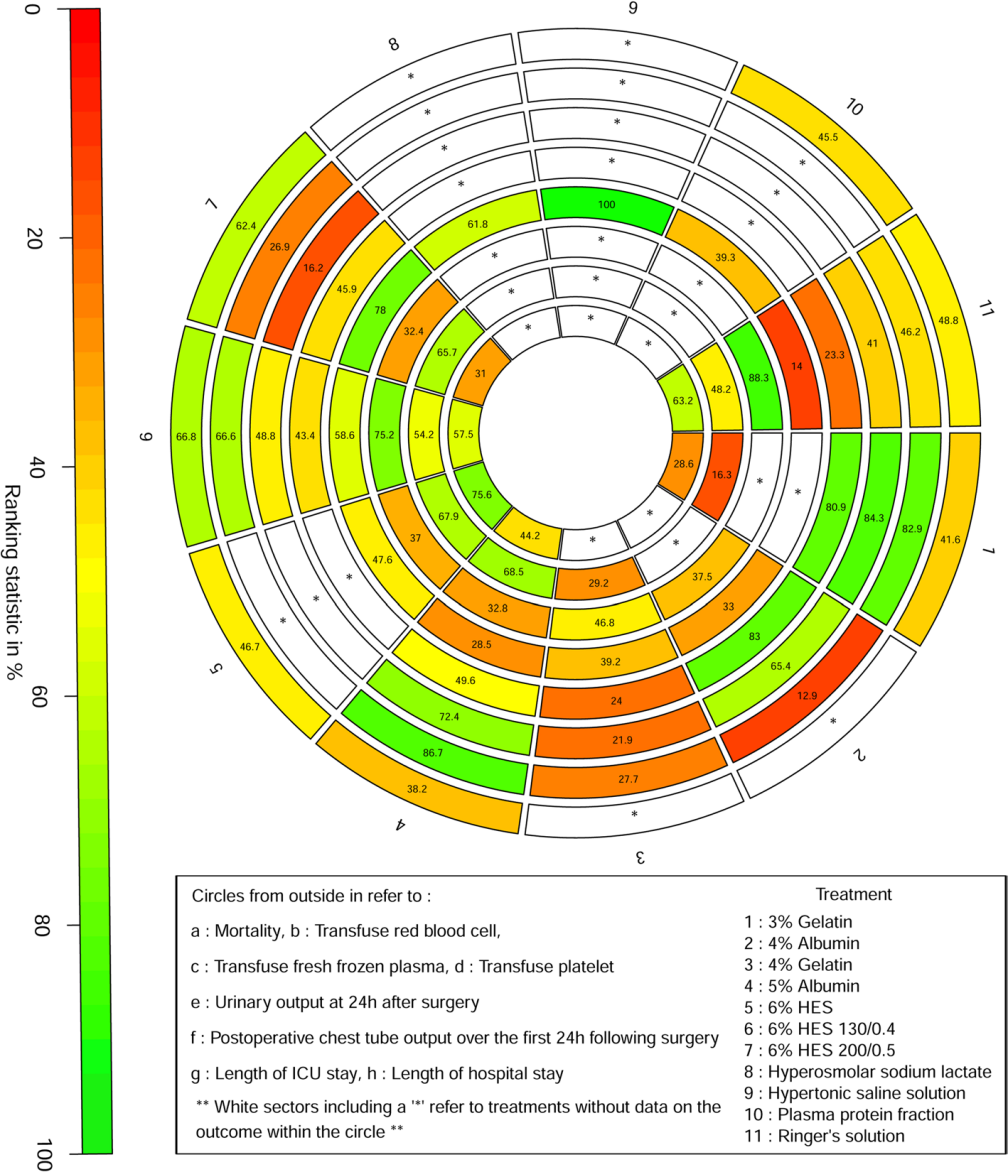
Ranking of perioperative fluid therapy for all outcomes

A total of eight studies included relevant data on mortality. Figure 2A shows the qualified network diagram of mortality for seven fluids, namely, 3% gelatine, 5% albumin, 6% HES, 6% HES 130/0.4, 6% HES 200/0.5, plasma protein fraction, and Ringer’s solution. All mortality data are presented in Table 2. None of the results, including network comparisons and direct comparisons, were statistically significant. Figure 3 shows that 6% HES 130/0.4 (66.8%) was associated with the lowest mortality rate, followed by 6% HES 200/0.5 (62.4%), while 5% albumin (38.2%) was associated with the highest mortality rate, followed by 3% gelatine (41.6%).

**Table 2:**
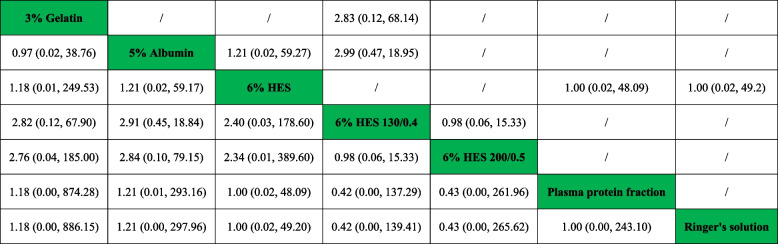
Network and direct comparison results for mortality

Comparisons between perioperative fluid therapy should be read from left to right, and the results are all comparisons between treatments defined on the top left and treatments defined on the bottom right. The table is divided into lower left and upper right sections with perioperative fluid therapy as the dividing line. The lower left part represents the network comparison results, and the upper right part represents the direct comparison results. For comparison results, when relative risk (RR) < 1, tended to define treatment on the left, when RR > 1, treatment tends to be defined to the lower right. Significant results are in bold and underline, and “/” means that the results are not available. *HES* hydroxyethyl starch

#### Number of patients who required transfusion of RBCs

A total of seven studies included relevant data on the number of patients who required transfusion of RBCs. Figure 2B shows the qualified network diagram of patients requiring RBC transfusion for seven fluids, namely, 3% gelatine, 4% albumin, 4% gelatine, 5% albumin, 6% HES 130/0.4, 6% HES 200/0.5, and Ringer’s solution. All results for the number of patients who required transfusion of RBCs are shown in Table 3. In the network results, significant differences were observed in the comparisons of 3% gelatine vs. 6% HES 200/0.5 (RR = 0.42, 95% CI = 0.20 ~ 0.90); 4% albumin vs. 5% albumin (RR = 3.47, 95% CI = 1.07 ~ 11.25); 4% gelatine vs. 5% albumin (RR = 2.46, 95% CI = 1.10 ~ 5.48); 5% albumin vs. 6% HES 200/0.5 (RR = 0.40, 95% CI = 0.22 ~ 0.74); and 6% HES 130/0.4 vs. 6% HES 200/0.5 (RR = 0.51, 95% CI = 0.28 ~ 0.92). Figure 3 shows that 5% albumin (86.7%) was associated with the lowest number of patients who required RBC transfusion, followed by 3% gelatine (82.9%), while 4% albumin (12.9%) was associated with the highest number of patients who required RBC transfusion, followed by 6% HES 200/0.5 (26.9%).

**Table 3:**
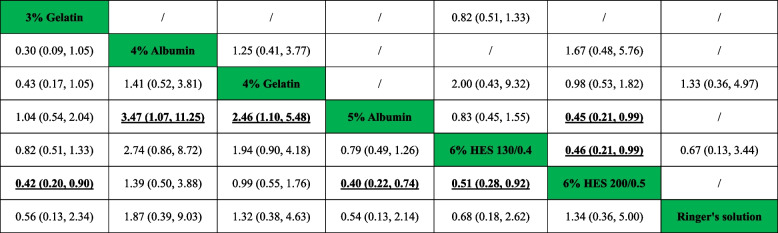
Network and direct comparison results for transfuse red blood cell

Comparisons between perioperative fluid therapy should be read from left to right, and the results are all comparisons between treatments defined on the top left and treatments defined on the bottom right. The table is divided into lower left and upper right sections with perioperative fluid therapy as the dividing line. The lower left part represents the network comparison results, and the upper right part represents the direct comparison results. For comparison results, when relative risk (RR) < 1, tended to define treatment on the left, when RR > 1, treatment tends to be defined to the lower right. Significant results are in bold and underline, and “/” means that the results are not available. *HES* hydroxyethyl starch

#### Number of patients who required transfusion of FFP

A total of seven studies included relevant data on the number of patients who required a transfuse of FFP. Figure 2C shows the qualified network diagram of the number of patients who required FFP transfusion for seven fluids, namely, 3% gelatine, 4% albumin, 4% gelatine, 5% albumin, 6% HES 130/0.4, 6% HES 200/0.5, and Ringer’s solution. All results for the number of patients who required FFP transfusion are shown in Supplementary Table 2. In the network results, significant differences were observed in comparisons of 3% gelatine vs. 4% gelatine (RR = 0.21, 95% CI = 0.07 ~ 0.69); 3% gelatine vs. 6% HES 200/0.5 (RR = 0.20, 95% CI = 0.06 ~ 0.63); 5% albumin vs. 6% HES 200/0.5 (RR = 0.32, 95% CI = 0.13 ~ 0.74); 4% gelatine vs. 5% albumin (RR = 3.00, 95% CI = 1.22 ~ 7.34); 4% gelatine vs. 6% HES 200/0.4 (RR = 1.62, 95% CI = 1.04 ~ 2.50); and 6% HES 130/0.4 vs. 6% HES 200/0.5 (RR = 0.59, 95% CI = 0.42 ~ 0.83). Figure 3 shows that 3% gelatine (84.3%) was associated with the lowest number of people who required FFP transfusion, followed by 5% albumin (72.4%), while 6% HES 200/0.5 (16.2%) was associated with the highest number of people who required FFP transfusion, followed by 4% gelatine (21.9%).

#### Number of patients who required transfusion of PLA

A total of five studies included relevant data on the number of patients who required transfusion of PLA. Figure 2D shows the qualified network diagram of the number of patients who required transfusion of PLA for seven fluids, namely, 3% gelatine, 4% albumin, 4% gelatine, 5% albumin, 6% HES 130/0.4, 6% HES 200/0.5, and Ringer’s solution. All results for the number of patients who required transfusion of PLA are shown in Supplementary Table 3. Figure 3 shows that 4% albumin (83.0%) was associated with the lowest number of patients who required PLA transfusion, followed by 3% gelatine (80.9%), while Ringer’s solution (23.3%) was associated with the highest number, followed by 4% gelatine (24.0%).

#### Acute kidney injury

Due to the small number of included studies, the outcome of acute kidney injury could not form a network link; therefore, only the traditional meta-analysis was performed. Only one study, including 6% HES 130/0.4 and 5% albumin, reported the risk of AKI. Compared with 6% HES 130/0.4, 5% albumin did not increase the incidence of risk of AKI (RR = 1.25, 95% CI = 0.99 ~ 1.58) in direct comparisons.

#### Serum creatinine

Due to the small number of included studies, the outcome of serum creatinine could not form a network link; therefore, only the traditional meta-analysis was performed. Compared with 6% HES in postoperative day 1, 4% gelatin did reduce serum creatinine (MD = 0.25, 95% CI = 0.15 ~ 0.35), while 5% albumin did not (MD =  − 0.10, 95% CI =  − 0.21 ~ 0.01). Compared with 6% HES 130/0.4 in postoperative day 1, 6% HES 200/0.5 did not reduce serum creatinine (SMD = 0.16, 95% CI =  − 0.13 ~ 0.46). Compared with Ringer’s solution in postoperative day 1, 6% HES did reduce serum creatinine (MD =  − 0.26, 95% CI =  − 0.36 to approximately − 0.16) while 4% gelatin did not (MD =  − 0.01, 95% CI =  − 0.13 ~ 0.10). Compared with 6% HES in postoperative day 7, 5% albumin did not reduce serum creatinine (MD =  − 0.10, 95% CI =  − 0.20 ~ 0.002). Compared with 6% HES 130/0.4 in postoperative day 7, 6% HES 200/0.5 did not reduce serum creatinine (MD = 0.10, 95% CI =  − 0.07 ~ 0.27).

#### Serum microglobulin

Due to the small number of included studies, the outcome of serum microglobulin could not form a network link; therefore, only the traditional meta-analysis was performed. Only one study, including 6% HES and 5% albumin, reported blood urea nitrogen. Compared with 6% HES, 5% albumin did not reduce serum microglobulin in postoperative day 1 (MD =  − 0.40, 95% CI =  − 0.91 ~ 0.11) and postoperative day 7 (MD =  − 0.30, 95% CI =  − 0.86 ~ 0.26).

#### Blood urea nitrogen

Due to the small number of included studies, the outcome of blood urea nitrogen could not form a network link; therefore, only the traditional meta-analysis was performed. Only one study, including 6% HES and 5% albumin, reported blood urea nitrogen. Compared with 6% HES, 5% albumin did not reduce blood urea nitrogen in postoperative day 1 (MD =  − 1.20, 95% CI =  − 3.40 ~ 1.00) and postoperative day 7 (MD = 0.40, 95% CI =  − 2.09 ~ 2.89).

#### Urinary output at 24 h after surgery

A total of 13 studies included relevant data on the urinary output at 24 h after surgery. Figure 2E presents the qualified network diagram of the urinary output at 24 h after surgery for 10 fluids, namely, 4% albumin, 4% gelatine, 5% albumin, 6% HES, 6% HES 130/0.4, 6% HES 200/0.5, hyperosmolar sodium lactate, hypertonic saline solution, plasma protein fraction, and Ringer’s solution. All results for the urinary output at 24 h after surgery are shown in Table 4. In the network results, statistical significance was observed for comparisons of 4% albumin vs. 6% HES 200/0.5 (MD =  − 445.24, 95% CI =  − 818.46 to approximately − 72.02); 4% albumin vs. hypertonic saline solution (MD =  − 2050.87, 95% CI =  − 2853.20 to approximately − 1248.54); 4% gelatine vs. hypertonic saline solution (MD =  − 2016.11, 95% CI =  − 2814.76 to approximately − 1217.46); 5% albumin vs. hypertonic saline solution (MD =  − 2143.29, 95% CI =  − 3192.41 to approximately − 1094.17); 6% HES vs. hypertonic saline solution (MD =  − 1942.96, 95% CI =  − 2865.06 to approximately − 1020.87); 6% HES 130/0.4 vs. hypertonic saline solution (MD =  − 1843.47, 95% CI =  − 2693.54 to approximately − 993.40); 6% HES 200/0.5 vs. hypertonic saline solution (MD =  − 1605.63, 95% CI =  − 2472.83 to approximately − 738.43); hyperosmolar sodium lactate vs. hypertonic saline solution (MD =  − 1757.55, 95% CI =  − 2879.35 to approximately − 635.75); hypertonic saline solution vs. plasma protein fraction (MD = 2028.84, 95% CI = 938.99 ~ 3118.69); 6% HES 200/0.5 vs. Ringer’s solution (MD = 699.84, 95% CI = 133.85 ~ 1205.83); and hypertonic saline solution vs. Ringer’s solution (MD = 2275.47, 95% CI = 1379.88 ~ 3171.06). Figure 3 shows that hypertonic saline solution (100.0%) was associated with the highest urinary output, followed by 6% HES 200/0.5 (78.0%), while Ringer’s solution (14.0%) was associated with the lowest urinary output, followed by 5% albumin (28.5%).

**Table 4:**
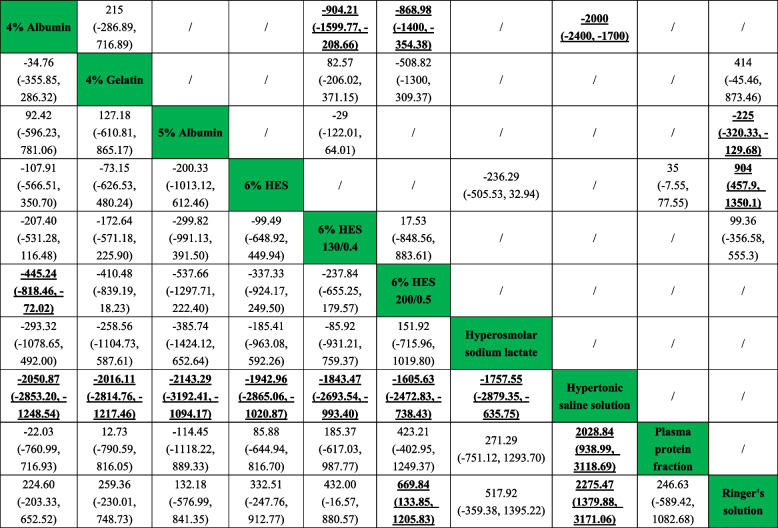
Network and direct comparison results for urinary output at 24 h after surgery

Comparisons between perioperative fluid therapy should be read from left to right, and the results are all comparisons between treatments defined on the top left and treatments defined on the bottom right. The table is divided into lower left and upper right sections with perioperative fluid therapy as the dividing line. The lower left part represents the network comparison results, and the upper right part represents the direct comparison results. For comparison results, when mean different (MD) > 0, tended to define treatment on the left, when MD < 0, treatment tends to be defined to the lower right. Significant results are in bold and underline, and “/” means that the results are not available. *HES* hydroxyethyl starch

#### Postoperative chest tube output over the first 24 h following surgery

A total of 13 studies included relevant data on the postoperative chest tube output over the first 24 h following surgery. Figure 2F shows the qualified network diagram of the postoperative chest tube output over the first 24 h following surgery for seven fluids, namely, 4% albumin, 4% gelatine, 5% albumin, 6% HES, 6% HES 130/0.4, 6% HES 200/0.5, and Ringer’s solution. All results for the postoperative chest tube output over the first 24 h following surgery are shown in Supplementary Table 4. None of the network results were statistically significant. Figure 3 shows that Ringer’s solution (88.3%) was associated with the lowest chest tube output within 24 h after surgery, followed by 6% HES 130/0.4 (75.2%), while 6% HES 200/0.5 (32.4%) was associated with the highest chest tube output within 24 h after surgery, followed by 5% albumin (32.8%).

#### Length of ICU stay

A total of six studies included relevant data on the length of ICU stay. Figure 2G shows the qualified network diagram of the length of ICU stay for seven fluids, namely, 3% gelatine, 4% gelatine, 5% albumin, 6% HES, 6% HES 130/0.4, 6% HES 200/0.5, and Ringer’s solution. All results for the length of ICU stay are shown in Supplementary Table 5. In the network results, statistical significance was observed for the comparisons of 4% gelatine vs. 6% HES (MD = 0.08, 95% CI = 0.05 ~ 0.11); 3% gelatine vs. 6% HES 130/0.4 (MD = 0.79, 95% CI = 0.59 ~ 0.99); 3% gelatine vs. 6% HES 200/0.5 (MD = 0.89, 95% CI = 0.57 ~ 1.22); 4% gelatine vs. Ringer’s solution (MD = 0.04, 95% CI = 0.01 ~ 0.07); 6% HES vs. Ringer’s solution (MD =  − 0.04, 95% CI =  − 0.07 to approximately − 0.01). Figure 3 shows that 5% albumin (68.5%) was associated with the lowest length of ICU stay, followed by 6% HES (67.9%), while 3% gelatine (16.3%) was associated with the highest length of ICU stay, followed by 4% gelatine (29.2%).

#### Length of hospital stay

A total of four studies included relevant data on the length of hospital stay. Figure 2H shows the qualified network diagram of the length of hospital stay for six fluids, namely, 3% gelatine, 5% albumin, 6% HES, 6% HES 130/0.4, 6% HES 200/0.5, and Ringer’s solution. All results for the length of hospital stay are shown in Supplementary Table 6. The network meta-analyses showed statistically significant results for none of the comparisons. Figure 3 shows that 6% HES (75.6%) was associated with the lowest length of hospital stay, followed by Ringer’s solution (63.2%), while 3% gelatine (28.6%) was associated with the highest length of hospital stay, followed by 6% HES 200/0.5 (31.0%).

#### Test of inconsistency

The results of the test of inconsistency for all outcomes are listed in Supplementary Tables 7–14. Supplementary Tables 11 and 12 show inconsistencies in the results related to urinary output at 24 h after surgery and postoperative chest tube output over the first 24 h following surgery. Other results of the test of inconsistency were not found inconsistency in Supplementary Tables 7–10, 13–14.

#### Publication bias

None of the outcomes showed publication bias, as reported in Supplementary Figs. 1–8.

## Discussion

To the best of our knowledge, this was the first NMA to comprehensively analysis the current available data on which kind of fluid (colloids or crystalloids) was more preferred in the perioperative period for cardiac surgery. This network meta-analysis of the effects of various intravenous injection fluids on mortality in cardiac surgery patients showed that cardiac surgery patients receiving 5% albumin in the perioperative period had the highest mortality rate. A study including 17,742 cardiac surgery patients also showed that perioperative use of albumin was associated with a significantly increased risk of 30-day and 6-month mortality (Ryhammer et al. 2017). The safety of the new generation of starches has been reported to be significantly better than that of the older starches (Jacob et al. 2014).

Perioperative transfusions of blood products such as RBCs, FFP, and PLA were used to correct blood volume loss and replenish the colloidal component of the blood to prevent and treat bleeding attributable to the complex coagulation dysfunction caused by reduced coagulation factors and reduced platelet count, thereby restoring and maintaining the coagulation function of the body and reducing bleeding (Stanworth et al. 2013; Kor et al. 2010). Regarding gelatine, a recent study found an increased need for both red blood cell and platelet transfusion in the gelatine group (Koponen et al. 2020). Similarly, the results of our study also suggest that patients receiving 4% gelatine require more transfusion of blood products. According to SUCRA results, 5% albumin was associated with the lowest number of patients who required RBC transfusion, while 4% albumin with the highest. In the effects of osmotic, the net movement of water will be towards the higher solute concentration, that 5% albumin will absorb more RBCs consequently the patients will require less RBCs compared with 4% albumin. An identical principle can be applied to explain FFP requirements for 3% gelatine vs. 4% gelatine. These findings run counter to the clinical practice, which might owe to the scarce literature that the quality of the collected data should be treated with caution.

AKI is one of the important indicators for postoperative patients, especially for kidney injury (Hobson et al. 2015). In addition, serum creatinine, serum microglobulin, blood urea nitrogen, and other indicators also can reflect the function of the patients with kidney damage (Lopez-Giacoman and Madero 2015; Lu et al. 2018; Seki et al. 2019). Several studies have also indicated that perioperative use of HES during cardiac surgery was not associated with increased AKI (Vives et al. 2016; Morath et al. 2021; Nagore et al. 2021). This study also found that 6% HES 130/0.4 performed equivalent to 5% albumin in risk of AKI for adult cardiac surgery based on a single study, and evidence on the performance of other perioperative fluids on the risk of AKI remains lacking. In addition, the evidences supported patients with perioperative 6% input hypothesis of serum creatinine level in postoperative day 1 was higher than 4% gelatin and Ringer’s solution, other types of perioperative fluid, including 6% HES 130/0.4, 6% HES 200/0.5, 6% HES, and 5% albumin, showed similar serum creatinine levels. Performance in serum microglobulin and blood urea nitrogen levels was comparable on the first and seventh days after using 6% HES with 5% albumin as the perioperative fluid. More importantly, due to the small number of studies included in the above outcomes, which may lead to imprecise results, the outcomes of kidney injury needed to be verified by larger samples of high-quality RCTs.

Urine output at 24 h postoperatively can be a useful predictor of early clinical outcome, and urine output and urine are closely related to renal function (Song et al. 2016). Urine output can identify acute kidney damage sooner than serum creatinine (Willner et al. 2021), which was considered a late biomarker. Furthermore, this study also found a smaller postoperative 24 h urinary output with Ringer’s solution versus HES. Related studies have shown that the use of 6% HES is not only better than Ringer’s solution in terms of volume expansion after CABG but also has a better short-term effect on renal function than Ringer's solution (Alavi et al. 2012).

Both crystalloids and colloids have their advantages and disadvantages, and their application should be weighed depending on the characteristics of each situation. This study used a network meta-analysis to compare and rank the effectiveness of different fluid therapy solutions in the perioperative management of cardiac surgery, providing a more valuable reference for updating the STS/SCA/AmSECT/SABM clinical practice guidelines on patient blood management recommendations.

## Limitations

This study had some limitations. Firstly, different studies used different infusion doses, which may have affected the results. Secondly, patients underwent different types of cardiac surgery, which may have led to differences in the effects of fluids on patients. Thirdly, due to the small number of included studies for the outcome, including AKI, serum creatinine, serum microglobulin, and blood urea nitrogen, could not form a network link, which also leads to only performing a traditional meta-analysis. Therefore, this research still needs to be a large sample of high-quality research to fill the end of the relevant safety data further. Finally, due to the exclusion of Boldt’s academic HES data and the retracted studies, some perioperative fluids such as 0.9% NaCl, plasma-lyte 148, and 7.2% NaCl plus 6% HES 200/0.5 could not be evaluated separately from the main network meta-analysis and further contributing to the lack of data included in studies, which limited the potential for extrapolation of the evidence.

## Conclusions

This study showed that 3% gelatin and 5% albumin can reduce the transfuse RBC and FFP. In addition, the use of hypertonic saline solution can increase urine output, and 5% albumin and 6% HES can shorten the length of ICU stay. However, none of the perioperative fluids showed an objective advantage in various outcomes, including mortality, transfuse PLA, postoperative chest tube output over the first 24 h following surgery, and length of hospital stay. The reliable and sufficient evidences on the injury of the kidney, including AKI, serum creatinine, serum microglobulin, and blood urea nitrogen, was still lacking. In general, perioperative fluids had advantages and disadvantages, and there were no evidences to support the recommendation of the optimal perioperative fluid for cardiac surgery. Clinicians should choose the type of perioperative fluid according to the actual condition of the cardiac surgery patient’s body.

### Supplementary Information

Additional file1: Supplementary materials.

## Data Availability

No datasets were generated or analysed during the current study.
