# Supplementary material for: Perioperative fluid management for adult cardiac surgery: network meta-analysis pooling on twenty randomised controlled trials
Source: Perioper Med (Lond). 2024 Jul 20;13:76. doi: 10.1186/s13741-024-00440-5 (PMC11264963; doi:10.1186/s13741-024-00440-5)

**Supplementary Materials**

**Supplementary Method 1** Search strategy

**Supplementary Table 1** Risk of bias for included studies

**Supplementary Table 2** Network and direct comparison results for transfuse fresh frozen plasma

**Note:** Comparisons between perioperative fluid therapy should be read from left to right, and the results are all comparisons between treatments defined on the top left and treatments defined on the bottom right. The table is divided into lower left and upper right sections with perioperative fluid therapy as the dividing line. The lower left part represents the network comparison results, and the upper right part represents the direct comparison results. For comparison results, when relative risk (RR) <1, tended to define treatment on the left, when RR >1, treatment tends to be defined to the lower right. Significant results are in bold and underline, and "/" means that the results are not available. HES: hydroxyethyl starch.

**Supplementary Table 3** Network and direct comparison results for transfuse platelet

**Note:** Comparisons between perioperative fluid therapy should be read from left to right, and the results are all comparisons between treatments defined on the top left and treatments defined on the bottom right. The table is divided into lower left and upper right sections with perioperative fluid therapy as the dividing line. The lower left part represents the network comparison results, and the upper right part represents the direct comparison results. For comparison results, when relative risk (RR) <1, tended to define treatment on the left, when RR >1, treatment tends to be defined to the lower right. Significant results are in bold and underline, and "/" means that the results are not available. HES: hydroxyethyl starch.

**Supplementary Table 4** Network and direct comparison results for postoperative chest tube output over the first 24h following surgery

**Note:** Comparisons between perioperative fluid therapy should be read from left to right, and the results are all comparisons between treatments defined on the top left and treatments defined on the bottom right. The table is divided into lower left and upper right sections with perioperative fluid therapy as the dividing line. The lower left part represents the network comparison results, and the upper right part represents the direct comparison results. For comparison results, when mean different (MD) <0, tended to define treatment on the left, when MD >0, treatment tends to be defined to the lower right. Significant results are in bold and underline, and "/" means that the results are not available. HES: hydroxyethyl starch.

**Supplementary Table 5** Network and direct comparison results for length of ICU stay

**Note:** Comparisons between perioperative fluid therapy should be read from left to right, and the results are all comparisons between treatments defined on the top left and treatments defined on the bottom right. The table is divided into lower left and upper right sections with perioperative fluid therapy as the dividing line. The lower left part represents the network comparison results, and the upper right part represents the direct comparison results. For comparison results, when mean different (MD) <0, tended to define treatment on the left, when MD >0, treatment tends to be defined to the lower right. Significant results are in bold and underline, and "/" means that the results are not available. HES: hydroxyethyl starch.

**Supplementary Table 6** Network and direct comparison results for length of hospital stay

**Note:** Comparisons between perioperative fluid therapy should be read from left to right, and the results are all comparisons between treatments defined on the top left and treatments defined on the bottom right. The table is divided into lower left and upper right sections with perioperative fluid therapy as the dividing line. The lower left part represents the network comparison results, and the upper right part represents the direct comparison results. For comparison results, when mean different (MD) <0, tended to define treatment on the left, when MD >0, treatment tends to be defined to the lower right. Significant results are in bold and underline, and "/" means that the results are not available. HES: hydroxyethyl starch.

**Supplementary Table 7** Test inconsistency for mortality

**Supplementary Table 8** Test inconsistency for transfuse red blood cell

**Supplementary Table 9** Test inconsistency for transfuse fresh frozen plasma

**Supplementary Table 10** Test inconsistency for transfuse platelet

**Supplementary Table 11** Test inconsistency for urinary output at 24h after surgery

**Supplementary Table 12** Test inconsistency for postoperative chest tube output over the first 24h following surgery

**Supplementary Table 13** Test inconsistency for length of ICU stay

**Supplementary Table 14** Test inconsistency for length of hospital stay

**Supplementary Figure 1** Funnel plot for mortality

**Supplementary Figure 2** Funnel plot for transfuse red blood cell

**Supplementary Figure 3** Funnel plot for transfuse fresh frozen plasma

**Supplementary Figure 4** Funnel plot for transfuse platelet

**Supplementary Figure 5** Funnel plot for urinary output at 24h after surgery

**Supplementary Figure 6** Funnel plot for postoperative chest tube output over the first 24h following surgery

**Supplementary Figure 7** Funnel plot for length of ICU stay

**Supplementary Figure 8** Funnel plot for length of hospital stay

**Supplementary Method 1** Search strategy

**1. Ovid MEDLINE(R) ALL 1946 to February 15, 2023**

1. Thoracic Surgery.mp.
2. Thoracic Surgical Procedures.mp.
3. Cardiac Surgical Procedures.mp.
4. exp heart surgery
5. Cardiac Surgery.mp.
6. Coronary Artery Bypass.mp.
7. (heart OR cardiac).ti,ab.
8. #1 OR #2 OR #3 OR #4 OR #5 OR #6 OR #7
9. Plasma Volume.mp.
10. Fluid Therapy.mp.
11. Colloids.mp.
12. Rehydration Solutions.mp.
13. Isotonic Solutions.mp.
14. Serum.mp.
15. Plasma.mp.
16. Plasma Substitutes.mp.
17. Albumin.mp.
18. Serum Albumin.mp.
19. Hydroxyethyl Starch Derivatives.mp.
20. Hetastarch.mp.
21. HAES-steril.mp.
22. Hydroxyethyl Starch.mp.
23. Ringer Lactate.mp.
24. Hartmanns Solution.mp.
25. Priming.mp.
26. Prime.mp.
27. (colloid* OR hydrocolloid* OR crystalloid* OR albumin* OR albumen* OR plasma OR starch* OR dextran* OR gelofus* OR hemaccel* OR haemaccel* OR serum OR hetastarch OR isotonic OR ringer* OR gelatin* OR gentran* OR pentastarch* OR pentaspan* OR hartman OR sodium OR potassium OR saline OR prime OR priming OR Hetastarch OR Ringer).ti,ab.
28. ((Isotonic adj1 saline adj1 solution*) OR (Blood adj1 substitut*) OR (blood adj1 expan*) OR (plasma adj1 volume adj1 expan*) OR (volume adj1 expan*)).ti,ab.
29. #9 OR #10 OR #11 OR #12 OR #13 OR #14 OR #15 OR #16 OR #17 OR #18 OR #19 OR #20 OR #21 OR #22 OR #23 OR #24 OR #25 OR #26 OR #27 OR #28
30. #8 AND #29
31. Limit #30 to (english language and humans and randomized controlled trial)

**2. Embase <1974 to February 15, 2023>**

1. ‘Thoracic Surgery’/exp
2. ‘Thoracic Surgical Procedures’/exp
3. ‘Cardiac Surgical Procedures’/exp
4. ‘Heart surgery’/exp
5. ‘Cardiac Surgery’/exp
6. ‘Coronary Artery Bypass’/exp
7. (heart OR cardiac):ti,ab
8. #1 OR #2 OR #3 OR #4 OR #5 OR #6 OR #7
9. ‘Plasma Volume’/exp
10. ‘Fluid Therapy’/exp
11. ‘Colloids’/exp
12. ‘Rehydration Solutions’/exp
13. ‘Isotonic Solutions’/exp
14. ‘Serum’/exp
15. ‘Plasma’/exp
16. ‘Plasma Substitutes’/exp
17. ‘Albumin’/exp
18. ‘Serum Albumin’/exp
19. ‘Hydroxyethyl Starch Derivatives’/exp
20. ‘Hetastarch’/exp
21. ‘HAES-steril’/exp
22. ‘Hydroxyethyl Starch’/exp
23. ‘Ringer Lactate’/exp
24. ‘Hartmanns Solution’/exp
25. ‘Priming’/exp
26. ‘Prime’/exp
27. (colloid* OR hydrocolloid* OR crystalloid* OR albumin* OR albumen* OR plasma OR starch* OR dextran* OR gelofus* OR hemaccel* OR haemaccel* OR serum OR hetastarch OR isotonic OR ringer* OR gelatin* OR gentran* OR pentastarch* OR pentaspan* OR hartman OR sodium OR potassium OR saline OR prime OR priming OR Hetastarch OR Ringer):ti,ab
28. #9 OR #10 OR #11 OR #12 OR #13 OR #14 OR #15 OR #16 OR #17 OR #18 OR #19 OR #20 OR #21 OR #22 OR #23 OR #24 OR #25 OR #26 OR #27
29. #8 AND #28
30. #29 AND [randomized controlled trial]/lim AND [english]/lim AND [humans]/lim AND ([embase]/lim OR [embase classic]/lim) AND [article]/lim

**3. Cochrane Central Register of Controlled Trials <** **February 15, 2023>**

1. MeSH descriptor: [Thoracic Surgery] explode all trees
2. MeSH descriptor: [Thoracic Surgical Procedures] explode all trees
3. MeSH descriptor: [Thoracic Surgery, Video-Assisted] explode all trees
4. MeSH descriptor: [Cardiac Surgical Procedures] explode all trees
5. MeSH descriptor: [Thoracic Surgery] explode all trees
6. MeSH descriptor: [Coronary Artery Bypass] explode all trees
7. (Heart or Cardiac):ti,ab
8. #1 or #2 or #3 or #4 or #5 or #6 or #7
9. MeSH descriptor: [Plasma Volume] explode all trees
10. MeSH descriptor: [Fluid Therapy] explode all trees
11. MeSH descriptor: [Colloids] explode all trees
12. MeSH descriptor: [Hetastarch] explode all trees
13. MeSH descriptor: [Hydroxyethyl Starch] explode all trees
14. MeSH descriptor: [Rehydration Solutions] explode all trees
15. MeSH descriptor: [Isotonic Solutions] explode all trees
16. MeSH descriptor: [Serum] explode all trees
17. MeSH descriptor: [Plasma] explode all trees
18. MeSH descriptor: [Plasma Substitutes] explode all trees
19. MeSH descriptor: [Albumins] explode all trees
20. MeSH descriptor: [Serum Albumin] explode all trees
21. MeSH descriptor: [Hartmanns Solution] explode all trees
22. (colloid* or hydrocolloid* or crystalloid* or albumin* or albumen* or plasma or starch* or dextran* or gelofus* OR hemaccel* or haemaccel* or serum or hetastarch or isotonic or ringer* or gelatin* or gentran* or pentastarch* or pentaspan* or hartman or sodium or potassium or saline or priming or prime):ti,ab
23. #9 or #10 or #11 or #12 or #13 or #14 or #15 or #16 or #17 or #18 or #19 or #20 or #22
24. #8 and #23 in Trials

**Supplementary Table 1** Risk of bias for included studies

| **Study** | **Year** | **Random sequence generation**  **(Selection bias)** | **Allocation concealment**  **(Selection bias)** | **Blinding of participants and personnel**  **(Performance bias)** | **Blinding of outcome assessment**  **(Detection bias)** | **Selective reporting (Reporting bias)** | **Incomplete outcome data (Attrition bias)** | **Other bias** |
| --- | --- | --- | --- | --- | --- | --- | --- | --- |
| Alavi | 2012 | Low Risk | Low Risk | Low Risk | Low Risk | Low Risk | High Risk | Low Risk |
| Belcher | 1983 | Low Risk | Low Risk | Low Risk | Unclear | Low Risk | Low Risk | Unclear |
| Boom | 2013 | Low Risk | Low Risk | Low Risk | Low Risk | Low Risk | Low Risk | Low Risk |
| Diehl | 1982 | Low Risk | Low Risk | Low Risk | Unclear | Low Risk | Low Risk | Unclear |
| Duncan | 2020 | Low Risk | Low Risk | Low Risk | Low Risk | Low Risk | Unclear | Low Risk |
| Huet | 2000 | Low Risk | Low Risk | Low Risk | Low Risk | Low Risk | Low Risk | Unclear |
| Kasper | 2003 | Low Risk | Low Risk | Low Risk | Low Risk | Low Risk | Low Risk | Unclear |
| Kuitunen | 2007 | Low Risk | Low Risk | Low Risk | Low Risk | Low Risk | Low Risk | Unclear |
| Lee | 2021 | Low Risk | Low Risk | Low Risk | Low Risk | Low Risk | Unclear | Low Risk |
| Linden | 2005 | Low Risk | Low Risk | Low Risk | Low Risk | Low Risk | Low Risk | Unclear |
| Mazhar | 1997 | Low Risk | Low Risk | Low Risk | Low Risk | Low Risk | Unclear | Low Risk |
| Munsch | 1988 | Low Risk | Low Risk | Low Risk | Unclear | Low Risk | Low Risk | Unclear |
| Niemi | 2008 | Low Risk | Low Risk | Low Risk | Low Risk | Low Risk | Low Risk | Unclear |
| Niemi | 2006 | Low Risk | Low Risk | Low Risk | Low Risk | Low Risk | Low Risk | Unclear |
| Öztürk a | 2014 | Low Risk | Low Risk | Low Risk | Low Risk | Unclear | Low Risk | Low Risk |
| Öztürk b | 2014 | Low Risk | Low Risk | Low Risk | Low Risk | Unclear | Low Risk | Low Risk |
| Schramko | 2009 | Low Risk | Low Risk | Low Risk | Low Risk | Low Risk | High Risk | Low Risk |
| Schramko | 2010 | Low Risk | Low Risk | Low Risk | Low Risk | Low Risk | High Risk | Low Risk |
| Schramko | 2010 | Low Risk | Low Risk | Low Risk | Low Risk | Low Risk | High Risk | Low Risk |
| Skhirtladze | 2013 | Low Risk | Low Risk | Low Risk | Low Risk | Unclear | Low Risk | Low Risk |

**Supplementary Table 2** Network and direct comparison results for transfuse fresh frozen plasma

| **3% Gelatin** | / | / | / | 0.34 (0.11, 1.02) | / | / |
| --- | --- | --- | --- | --- | --- | --- |
| 0.79 (0.03, 18.06) | **4% Albumin** | / | / | 0.33 (0.01, 7.58) | 0.33 (0.01, 7.58) | / |
| **0.21 (0.07, 0.69)** | 0.27 (0.01, 5.15) | **4% Gelatin** | / | 1.00 (0.02, 47.38) | 0.95 (0.72, 1.25) | 1.00 (0.02, 47.38) |
| 0.63 (0.17, 2.43) | 0.81 (0.04, 16.87) | **3.00 (1.22, 7.34)** | **5% Albumin** | 0.57 (0.26, 1.24) | / | / |
| 0.34 (0.11, 1.02) | 0.44 (0.02, 8.22) | **1.62 (1.04, 2.50)** | 0.54 (0.25, 1.18) | **6% HES 130/0.4** | **0.58 (0.41, 0.82)** | 1.00 (0.02, 47.38) |
| **0.20 (0.06, 0.63)** | 0.26 (0.01, 4.82) | 0.95 (0.72, 1.24) | **0.32 (0.13, 0.74)** | **0.59 (0.42, 0.83)** | **6% HES 200/0.5** | / |
| 0.27 (0.01, 9.11) | 0.34 (0.00, 29.26) | 1.27 (0.04, 36.19) | 0.42 (0.01, 13.22) | 0.79 (0.03, 22.38) | 1.34 (0.05, 38.19) | **Ringer's solution** |

**Note:** Comparisons between perioperative fluid therapy should be read from left to right, and the results are all comparisons between treatments defined on the top left and treatments defined on the bottom right. The table is divided into lower left and upper right sections with perioperative fluid therapy as the dividing line. The lower left part represents the network comparison results, and the upper right part represents the direct comparison results. For comparison results, when relative risk (RR) <1, tended to define treatment on the left, when RR >1, treatment tends to be defined to the lower right. Significant results are in bold and underline, and "/" means that the results are not available. HES: hydroxyethyl starch.

**Supplementary Table 3** Network and direct comparison results for transfuse platelet

| **3% Gelatin** | / | / | / | 0.35 (0.10, 1.27) | / | / |
| --- | --- | --- | --- | --- | --- | --- |
| 1.76 (0.07, 44.25) | **4% Albumin** | / | / | 0.20 (0.01, 3.85) | 0.20 (0.01, 3.85) | / |
| 0.12 (0.00, 3.45) | 0.07 (0.00, 4.92) | **4% Gelatin** | / | 3.00 (0.13, 68.26) | / | / |
| 0.39 (0.09, 1.59) | 0.22 (0.01, 4.47) | 3.30 (0.14, 79.11) | **5% Albumin** | 0.92 (0.51, 1.66) | / | / |
| 0.35 (0.10, 1.27) | 0.20 (0.01, 3.84) | 3.00 (0.13, 68.19) | 0.91 (0.51, 1.63) | **6% HES 130/0.4** | 1.00 (0.16, 6.20) | 0.33 (0.04, 3.04) |
| 0.35 (0.05, 2.76) | 0.20 (0.01, 3.84) | 3.00 (0.09, 100.78) | 0.91 (0.16, 5.04) | 1.00 (0.20, 5.00) | **6% HES 200/0.5** | / |
| 0.12 (0.00, 3.45) | 0.07 (0.00, 4.92) | 1.00 (0.12, 8.62) | 0.30 (0.01, 7.28) | 0.33 (0.01, 7.59) | 0.33 (0.01, 11.21) | **Ringer's solution** |

**Note:** Comparisons between perioperative fluid therapy should be read from left to right, and the results are all comparisons between treatments defined on the top left and treatments defined on the bottom right. The table is divided into lower left and upper right sections with perioperative fluid therapy as the dividing line. The lower left part represents the network comparison results, and the upper right part represents the direct comparison results. For comparison results, when relative risk (RR) <1, tended to define treatment on the left, when RR >1, treatment tends to be defined to the lower right. Significant results are in bold and underline, and "/" means that the results are not available. HES: hydroxyethyl starch.

**Supplementary Table 4** Network and direct comparison results for postoperative chest tube output over the first 24h following surgery

| **4% Albumin** | **-230**  **(-451.85, -8.15)** | / | / | **108.86**  **(41.01, 176.71)** | -49.26  (-225.17, 126.65) | / |
| --- | --- | --- | --- | --- | --- | --- |
| 10.58  (-76.29, 97.46) | **4% Gelatin** | / | / | 7.88  (-102.87, 118.63) | **-67.25**  **(-129.61, -4.90)** | 178  (-21.59, 377.59) |
| -10.83  (-114.32, 92.67) | -21.41  (-145.88, 103.06) | **5% Albumin** | -142.00  (-301.53, 17.53) | 96.07  (-17.38, 209.52) | / | **165**  **(123.12, 206.88)** |
| -8.89  (-154.33, 136.55) | -19.48  (-176.88, 137.92) | 1.93  (-143.46, 147.33) | **6% HES** | / | / | -34  (-158.15, 90.15) |
| 51.45  (-31.22, 134.12) | 40.87  (-51.40, 133.14) | 62.28  (-40.16, 164.71) | 60.34  (-85.86, 206.54) | **6% HES 130/0.4** | 0.00  (-86.11, 86.11) | 30  (-4.88, 64.88) |
| -8.46  (-101.34, 84.43) | -19.04  (-114.50, 76.41) | 2.37  (-128.31, 133.05) | 0.44  (-161.61, 162.48) | -59.91  (-159.08, 39.27) | **6% HES 200/0.5** | / |
| 83.24  (-28.05, 194.53) | 72.65  (-49.14, 194.45) | 94.06  (-20.37, 208.49) | 92.13  (-47.98, 232.25) | 31.79  (-70.18, 133.75) | 91.70  (-38.11, 221.50) | **Ringer's solution** |

**Note:** Comparisons between perioperative fluid therapy should be read from left to right, and the results are all comparisons between treatments defined on the top left and treatments defined on the bottom right. The table is divided into lower left and upper right sections with perioperative fluid therapy as the dividing line. The lower left part represents the network comparison results, and the upper right part represents the direct comparison results. For comparison results, when mean different (MD) <0, tended to define treatment on the left, when MD >0, treatment tends to be defined to the lower right. Significant results are in bold and underline, and "/" means that the results are not available. HES: hydroxyethyl starch.

**Supplementary Table 5** Network and direct comparison results for length of ICU stay

| **3% Gelatin** | / | / | / | **0.79 (0.59, 0.99)** | / | / |
| --- | --- | --- | --- | --- | --- | --- |
| 0.71 (-1.47, 2.90) | **4% Gelatin** | / | **0.08 (0.05, 0.11)** | / | / | **0.04 (0.01, 0.07)** |
| 0.83 (-1.36, 3.01) | 0.12 (-0.07, 0.31) | **5% Albumin** | -0.04 (-0.23, 0.15) | 0.00 (-0.31, 0.31) | / | 0.00 (-0.98, 0.98) |
| 0.79 (-1.39, 2.98) | **0.08 (0.05, 0.11)** | -0.04 (-0.22, 0.15) | **6% HES** | / | / | **-0.04 (-0.07, -0.01)** |
| **0.79 (0.59, 0.99)** | 0.08 (-2.10, 2.25) | -0.04 (-2.22, 2.14) | -0.00 (-2.18, 2.17) | **6% HES 130/0.4** | 0.10 (-0.16, 0.36) | 0.00 (-2.22, 2.22) |
| **0.89 (0.57, 1.22)** | 0.18 (-2.01, 2.37) | 0.06 (-2.13, 2.25) | 0.10 (-2.09, 2.29) | 0.10 (-0.15, 0.36) | **6% HES 200/0.5** | / |
| 0.75 (-1.43, 2.94) | **0.04 (0.01, 0.07)** | -0.08 (-0.27, 0.11) | **-0.04 (-0.07, -0.01)** | -0.04 (-2.21, 2.14) | -0.14 (-2.33, 2.05) | **Ringer's solution** |

**Note:** Comparisons between perioperative fluid therapy should be read from left to right, and the results are all comparisons between treatments defined on the top left and treatments defined on the bottom right. The table is divided into lower left and upper right sections with perioperative fluid therapy as the dividing line. The lower left part represents the network comparison results, and the upper right part represents the direct comparison results. For comparison results, when mean different (MD) <0, tended to define treatment on the left, when MD >0, treatment tends to be defined to the lower right. Significant results are in bold and underline, and "/" means that the results are not available. HES: hydroxyethyl starch.

**Supplementary Table 6** Network and direct comparison results for length of hospital stay

| **3% Gelatin** | / | / | 1.00 (-0.70, 2.70) | / | / |
| --- | --- | --- | --- | --- | --- |
| 1.00 (-2.82, 4.82) | **5% Albumin** | 1.00 (-0.07, 2.07) | 0.00 (-3.42, 3.42) | / | 1.00 (-6.70, 8.70) |
| 2.00 (-1.97, 5.96) | 1.00 (-0.07, 2.07) | **6% HES** | / | / | / |
| 1.00 (-0.70, 2.70) | 0.00 (-3.42, 3.42) | -1.00 (-4.58, 2.58) | **6% HES 130/0.4** | -1.00 (-3.46, 1.46) | 1.00 (-6.51, 8.51) |
| -0.00 (-2.99, 2.99) | -1.00 (-5.21, 3.21) | -2.00 (-6.34, 2.35) | -1.00 (-3.46, 1.46) | **6% HES 200/0.5** | / |
| 2.00 (-5.70, 9.70) | 1.00 (-6.70, 8.70) | 0.00 (-7.77, 7.77) | 1.00 (-6.51, 8.51) | 2.00 (-5.91, 9.91) | **Ringer's solution** |

**Note:** Comparisons between perioperative fluid therapy should be read from left to right, and the results are all comparisons between treatments defined on the top left and treatments defined on the bottom right. The table is divided into lower left and upper right sections with perioperative fluid therapy as the dividing line. The lower left part represents the network comparison results, and the upper right part represents the direct comparison results. For comparison results, when mean different (MD) <0, tended to define treatment on the left, when MD >0, treatment tends to be defined to the lower right. Significant results are in bold and underline, and "/" means that the results are not available. HES: hydroxyethyl starch.

**Supplementary Table 7** Test inconsistency for mortality

| **Side** | **Direct** | |  | **Indirect** | |  | **Difference** | | | **Tau** |
| --- | --- | --- | --- | --- | --- | --- | --- | --- | --- | --- |
|  | **Coef.** | **Std. Err.** |  | **Coef.** | **Std. Err.** |  | **Coef.** | **Std. Err.** | **P>z** |  |
| 3% Gelatin vs. 6% HES 130/0.4 | -1.04 | 1.623819 |  | -0.7069666 | 37.80761 |  | -0.3319265 | 37.84246 | 0.993 | 1.00E-08 |
| 5% Albumin vs. 6% HES | -0.1941365 | 1.983565 |  | -0.0540295 | 66.70177 |  | -0.140107 | 66.72471 | 0.998 | 6.71E-09 |
| 5% Albumin vs. 6% HES 130/0.4 | -1.07 | 0.9535394 |  | -1.145095 | 53.49636 |  | 0.0775041 | 53.50728 | 0.999 | 1.12E-09 |
| 6% HES vs. Plasma protein fraction | 1.96E-10 | 1.976047 |  | 0.3296259 | 200.0951 |  | -0.3296259 | 200.0854 | 0.999 | 7.59E-11 |
| 6% HES vs. Ringer's solution | 2.43E-10 | 1.987767 |  | 0.3296251 | 200.1149 |  | -0.3296251 | 200.105 | 0.999 | 5.05E-10 |
| 6% HES 130/0.4 vs. 6% HES 200/0.5 | 2.48E-02 | 1.405406 |  | 2.102488 | 141.4833 |  | -2.077657 | 141.4763 | 0.988 | 4.77E-09 |
| 3% Gelatin vs. 6% HES 130/0.4 | -1.04 | 1.623819 |  | -0.7069666 | 37.80761 |  | -0.3319265 | 37.84246 | 0.993 | 1.00E-08 |

**Supplementary Table 8** Test inconsistency for transfuse red blood cell

| **Side** | **Direct** | |  | **Indirect** | |  | **Difference** | | | **Tau** |
| --- | --- | --- | --- | --- | --- | --- | --- | --- | --- | --- |
|  | **Coef.** | **Std. Err.** |  | **Coef.** | **Std. Err.** |  | **Coef.** | **Std. Err.** | **P>z** |  |
| 3% Gelatin vs. 6% HES 130/0.4 | 0.194156 | 0.242806 |  | -0.26912 | 40.82975 |  | 0.463279 | 40.83047 | 0.991 | 1.68E-09 |
| 4% Albumin vs. 4% Gelatin | -0.22313 | 0.56273 |  | -0.97013 | 1.348162 |  | 0.747009 | 1.494732 | 0.617 | 5.40E-09 |
| 4% Albumin vs. 6% HES 200/0.5 | -0.51081 | 0.632454 |  | 0.236331 | 1.252015 |  | -0.74714 | 1.494732 | 0.617 | 7.09E-09 |
| 4% Gelatin vs. 6% HES 130/0.4 | -0.69312 | 0.785275 |  | -0.65024 | 0.452653 |  | -0.04288 | 0.906398 | 0.962 | 1.45E-08 |
| 4% Gelatin vs. 6% HES 200/0.5 | 0.018443 | 0.315466 |  | -0.02431 | 0.849705 |  | 0.042757 | 0.906378 | 0.962 | 4.43E-09 |
| 4% Gelatin vs. Ringer's solution | -0.28767 | 0.670818 |  | -0.20198 | 1.73286 |  | -0.08568 | 1.812759 | 0.962 | 1.02E-07 |
| 5% Albumin vs. 6% HES 130/0.4 | 0.239464 | 0.240618 |  | 0.154125 | 1.796419 |  | 0.085339 | 1.81247 | 0.962 | 2.31E-09 |
| 5% Albumin vs. 6% HES 200/0.5 | 0.816667 | 0.386702 |  | 1.183302 | 0.715264 |  | -0.36663 | 0.869333 | 0.673 | 1.58E-07 |
| 6% HES 130/0.4 vs. 6% HES 200/0.5 | 0.754202 | 0.388055 |  | 0.486547 | 0.644159 |  | 0.267656 | 0.810728 | 0.741 | 1.67E-08 |
| 6% HES 130/0.4 vs. Ringer's solution | 4.05E-01 | 0.83666 |  | 0.319638 | 1.500966 |  | 0.085827 | 1.812797 | 0.962 | 6.79E-08 |

**Supplementary Table 9** Test inconsistency for transfuse fresh frozen plasma

| **Side** | **Direct** | |  | **Indirect** | |  | **Difference** | | | **Tau** |
| --- | --- | --- | --- | --- | --- | --- | --- | --- | --- | --- |
|  | **Coef.** | **Std. Err.** |  | **Coef.** | **Std. Err.** |  | **Coef.** | **Std. Err.** | **P>z** |  |
| 3% Gelatin vs. 6% HES 130/0.4 | 1.072226 | 0.5572954 |  | 0.1836902 | 40.8093 |  | 0.8885354 | 40.8131 | 0.983 | 2.67E-07 |
| 4% Albumin vs. 6% HES 130/0.4 | 1.098713 | 1.594143 |  | 0.001712 | 2.254714 |  | 1.097001 | 2.227016 | 0.622 | 7.11E-09 |
| 4% Albumin vs. 6% HES 200/0.5 | 1.098607 | 1.594143 |  | 2.195672 | 2.254723 |  | -1.097065 | 2.22702 | 0.622 | 1.36E-08 |
| 4% Gelatin vs. 6% HES 130/0.4 | 0.0002549 | 1.968407 |  | -0.487109 | 0.224378 |  | 0.4873637 | 1.981156 | 0.806 | 3.42E-07 |
| 4% Gelatin vs. 6% HES 200/0.5 | 0.0512946 | 0.1389181 |  | 0.5390808 | 1.975901 |  | -0.487786 | 1.980779 | 0.805 | 1.84E-07 |
| 4% Gelatin vs. Ringer's solution | 0.0002076 | 1.968407 |  | -0.974846 | 3.43846 |  | 0.9750535 | 3.961739 | 0.806 | 2.71E-06 |
| 5% Albumin vs. 6% HES 130/0.4 | 0.616539 | 0.3994603 |  | 1.52756 | 141.4357 |  | -0.911021 | 141.4374 | 0.995 | 4.37E-07 |
| 6% HES 130/0.4 vs. 6% HES 200/0.5 | 0.5384152 | 0.1762032 |  | 0.0503013 | 1.972445 |  | 0.4881139 | 1.980298 | 0.805 | 1.22E-07 |
| 6% HES 130/0.4 vs. Ringer's solution | 1.23E-12 | 1.968502 |  | 0.9742083 | 3.43895 |  | -0.974208 | 3.962497 | 0.806 | 1.05E-08 |

**Supplementary Table 10** Test inconsistency for transfuse platelet

| **Side** | **Direct** | |  | **Indirect** | |  | **Difference** | | | **Tau** |
| --- | --- | --- | --- | --- | --- | --- | --- | --- | --- | --- |
|  | **Coef.** | **Std. Err.** |  | **Coef.** | **Std. Err.** |  | **Coef.** | **Std. Err.** | **P>z** |  |
| 3% Gelatin vs. 6% HES 130/0.4 | 1.041454 | 0.654219 |  | 0.175052 | 49.99762 |  | 0.866402 | 50.0019 | 0.986 | 1.26E-09 |
| 4% Albumin vs. 6% HES 130/0.4 | 1.609438 | 1.50831 |  | 0.473124 | 200.0189 |  | 1.136314 | 200.0359 | 0.995 | 6.08E-10 |
| 4% Albumin vs. 6% HES 200/0.5 | 1.609328 | 1.80959 |  | 1.598655 | 7262.511 |  | 0.010673 | 7262.511 | 1.000 | 0.999995 |
| 4% Gelatin vs. 6% HES 130/0.4 | -1.09861 | 1.594261 |  | 3.181406 | 200.0139 |  | -4.28002 | 200.0329 | 0.983 | 4.73E-09 |
| 5% Albumin vs. 6% HES 130/0.4 | 0.09469 | 0.296989 |  | 1.98809 | 141.3429 |  | -1.8934 | 141.3439 | 0.989 | 7.71E-09 |
| 6% HES 130/0.4 vs. 6% HES 200/0.5 | 1.80E-12 | 0.821584 |  | 1.136313 | 199.8268 |  | -1.13631 | 199.8285 | 0.995 | 2.31E-09 |
| 6% HES 130/0.4 vs. Ringer's solution | 1.098612 | 1.594261 |  | -3.18141 | 200.0632 |  | 4.280019 | 200.0762 | 0.983 | 2.33E-10 |

**Supplementary Table 11** Test inconsistency for urinary output at 24h after surgery

| **Side** | **Direct** | |  | **Indirect** | |  | **Difference** | | | **Tau** |
| --- | --- | --- | --- | --- | --- | --- | --- | --- | --- | --- |
|  | **Coef.** | **Std. Err.** |  | **Coef.** | **Std. Err.** |  | **Coef.** | **Std. Err.** | **P>z** |  |
| 4% Albumin vs. 4% Gelatin | -236.3244 | 485.7059 |  | 70.7211 | 177.0708 |  | -307.0455 | 517.6442 | 0.553 | 413.142 |
| 4% Albumin vs. 6% HES 130/0.4 | 944.0253 | 226.0533 |  | -82.96633 | 147.4457 |  | 1026.992 | 268.7722 | 0.000* | 280.7974 |
| 4% Albumin vs. 6% HES 200/0.5 | 901.8974 | 223.5678 |  | -16.89571 | 227.713 |  | 918.7931 | 315.8094 | 0.004* | 327.6606 |
| 4% Gelatin vs. 6% HES 130/0.4 | -21.42245 | 286.3013 |  | 382.0835 | 298.0613 |  | -403.5059 | 415.1397 | 0.331 | 412.1221 |
| 4% Gelatin vs. 6% HES 200/0.5 | 482.3164 | 326.9281 |  | 345.2224 | 310.4206 |  | 137.0939 | 451.0108 | 0.761 | 420.587 |
| 4% Gelatin vs. Hypertonic saline solution | 2032.5 | 458.891 |  | 1952.195 | 859.6801 |  | 80.30514 | 939.2841 | 0.932 | 420.5863 |
| 4% Gelatin vs. Ringer's solution | -355.625 | 365.423 |  | -179.6263 | 343.9272 |  | -175.9987 | 493.9109 | 0.722 | 417.2055 |
| 5% Albumin vs. 6% HES 130/0.4 | 25.40087 | 395.9782 |  | 1100.14 | 679.1108 |  | -1074.74 | 787.7244 | 0.172 | 393.1304 |
| 5% Albumin vs. Ringer's solution | 226.6295 | 373.2531 |  | -1414.049 | 717.6803 |  | 1640.678 | 810.3932 | 0.043* | 370.081 |
| 6% HES vs. Hyperosmolar sodium lactate | 236.2901 | 441.5961 |  | -79.04658 | 961.7442 |  | 315.3366 | 1040.298 | 0.762 | 419.6873 |
| 6% HES vs. Plasma protein fraction | -34.99999 | 420.2483 |  | -350.3366 | 952.131 |  | 315.3366 | 1040.298 | 0.762 | 419.6873 |
| 6% HES vs. Ringer's solution | -837.0324 | 450.8215 |  | 27.52544 | 375.791 |  | -864.5578 | 588.8156 | 0.142 | 393.3179 |
| 6% HES 130/0.4 vs. 6% HES 200/0.5 | 9.567407 | 263.0266 |  | 639.9582 | 348.7869 |  | -630.3908 | 435.6371 | 0.148 | 400.8304 |
| 6% HES 130/0.4 vs. Ringer's solution | -112.6366 | 249.6304 |  | -1179.272 | 392.9105 |  | 1066.636 | 467.7133 | 0.023* | 362.2957 |

**Note:** * indicated an inconsistency in this comparison.

**Supplementary Table 12** Test inconsistency for postoperative chest tube output over the first 24h following surgery

| **Side** | **Direct** | |  | **Indirect** | |  | **Difference** | | | **Tau** |
| --- | --- | --- | --- | --- | --- | --- | --- | --- | --- | --- |
|  | **Coef.** | **Std. Err.** |  | **Coef.** | **Std. Err.** |  | **Coef.** | **Std. Err.** | **P>z** |  |
| 4% Albumin vs. 4% Gelatin | 260.8314 | 119.1226 |  | -52.01498 | 42.02702 |  | 312.8464 | 126.6628 | 0.014* | 55.89644 |
| 4% Albumin vs. 6% HES 130/0.4 | -118.6627 | 58.3276 |  | -0.3706845 | 53.10556 |  | -118.292 | 78.11934 | 0.130 | 66.3534 |
| 4% Albumin vs. 6% HES 200/0.5 | -11.59502 | 58.4245 |  | 50.47388 | 78.10275 |  | -62.0689 | 96.84139 | 0.522 | 68.96685 |
| 4% Gelatin vs. 6% HES 130/0.4 | -21.10284 | 64.57125 |  | -69.84532 | 77.98327 |  | 48.74248 | 103.5699 | 0.638 | 75.25443 |
| 4% Gelatin vs. 6% HES 200/0.5 | 81.30602 | 60.47199 |  | -63.55246 | 69.38266 |  | 144.8585 | 91.73364 | 0.114 | 62.39994 |
| 4% Gelatin vs. 6% HES 200/0.5 | -149.8374 | 101.3502 |  | -31.1755 | 74.94677 |  | -118.6619 | 123.4484 | 0.336 | 69.63986 |
| 5% Albumin vs. 6% HES | 123.3236 | 96.54365 |  | -116.9914 | 87.5065 |  | 240.3149 | 126.9506 | 0.058 | 57.60013 |
| 5% Albumin vs. 6% HES 130/0.4 | -94.56001 | 60.75265 |  | 20.981 | 98.4141 |  | -115.541 | 116.622 | 0.322 | 70.64145 |
| 5% Albumin vs. Ringer's solution | -163.2404 | 61.98951 |  | 16.38831 | 85.50089 |  | -179.6287 | 105.5564 | 0.089 | 58.23509 |
| 6% HES vs. Ringer's solution | 34.08764 | 79.32968 |  | -288.3166 | 101.507 |  | 322.4042 | 132.5177 | 0.015* | 50.20412 |
| 6% HES 130/0.4 vs. 6% HES 200/0.5 | -1.29E-11 | 65.15618 |  | 140.1419 | 76.07137 |  | -140.1419 | 100.1608 | 0.162 | 68.15141 |
| 6% HES 130/0.4 vs. Ringer's solution | -28.62396 | 62.13476 |  | -43.06058 | 107.4309 |  | 14.43661 | 123.7596 | 0.907 | 74.87098 |

**Note:** * indicated an inconsistency in this comparison.

**Supplementary Table 13** Test inconsistency for length of ICU stay

| **Side** | **Direct** | |  | **Indirect** | |  | **Difference** | | | **Tau** |
| --- | --- | --- | --- | --- | --- | --- | --- | --- | --- | --- |
|  | **Coef.** | **Std. Err.** | | **Coef.** | **Std. Err.** | | **Coef.** | **Std. Err.** | **P>z** |  |
| 3% Gelatin vs. 6% HES 130/0.4 | -0.79 | 0.1022401 |  | 0.0034097 | 1.10749 |  | -0.7934097 | 1.112199 | 0.476 | 3.57E-06 |
| 4% Gelatin vs. 6% HES | -0.08 | 0.0138977 |  | 0.0800228 | 1.023359 |  | -0.1600228 | 1.023485 | 0.876 | 5.93E-07 |
| 4% Gelatin vs. Ringer's solution | -0.0400001 | 0.0143102 |  | -0.2000595 | 1.021678 |  | 0.1600594 | 1.02E+00 | 0.876 | 1.05E-07 |
| 5% Albumin vs. 6% HES | 0.0399997 | 0.0968789 |  | -0.0400006 | 0.5016827 |  | 0.0800003 | 5.11E-01 | 0.876 | 7.75E-07 |
| 5% Albumin vs. 6% HES 130/0.4 | -0.0002573 | 1.139524 |  | 0.1596019 | 1.35766 |  | -0.1598592 | 1.02E+00 | 0.876 | 0.0000588 |
| 5% Albumin vs. Ringer's solution | -0.0000107 | 0.5013348 |  | 0.08 | 0.0980929 |  | -0.0800108 | 5.11E-01 | 0.876 | 6.40E-06 |
| 6% HES vs. Ringer's solution | 0.04 | 0.015383 |  | -0.040021 | 0.5106759 |  | 0.0800209 | 0.5109076 | 0.876 | 1.02E-06 |
| 6% HES 130/0.4 vs. 6% HES 200/0.5 | -0.1006526 | 0.1303215 |  | 1.479345 | 141.1109 |  | -1.579998 | 1.41E+02 | 0.991 | 8.88E-08 |
| 6% HES 130/0.4 vs. Ringer's solution | 0.0002064 | 1.134397 |  | 0.1603625 | 1.359041 |  | -0.160156 | 1.02E+00 | 0.876 | 4.05E-07 |

**Supplementary Table 14** Test inconsistency for length of hospital stay

| **Side** | **Direct** | |  | **Indirect** | |  | **Difference** | | | **Tau** |
| --- | --- | --- | --- | --- | --- | --- | --- | --- | --- | --- |
|  | **Coef.** | **Std. Err.** |  | **Coef.** | **Std. Err.** |  | **Coef.** | **Std. Err.** | **P>z** |  |
| 3% Gelatin vs. 6% HES 130/0.4 | -1 | 0.865293 |  | -5.67E-07 | 57.73122 |  | -1 | 57.7377 | 0.986 | 5.98E-06 |
| 5% Albumin vs. 6% HES | -0.99999 | 0.547879 |  | 0.9986092 | 199.8512 |  | -1.9986 | 199.8505 | 0.992 | 0.001217 |
| 5% Albumin vs. 6% HES 130/0.4 | -4E-05 | 1.745144 |  | -1.199849 | 128.2539 |  | 1.199809 | 128.2759 | 0.993 | 0.000128 |
| 5% Albumin vs. Ringer's solution | -1.00013 | 3.926706 |  | -2.998953 | 1293.229 |  | 1.998822 | 1293.229 | 0.999 | 0.000853 |
| 6% HES 130/0.4 vs. 6% HES 200/0.5 | 1.000057 | 1.253753 |  | 2.999411 | 200.0708 |  | -1.99935 | 200.0668 | 0.992 | 0.000704 |
| 6% HES 130/0.4 vs. Ringer's solution | -0.99995 | 3.833661 |  | 0.9998196 | 1610.041 |  | -1.99977 | 1610.032 | 0.999 | 0.001336 |

**Supplementary Figure 1** Funnel plot for mortality


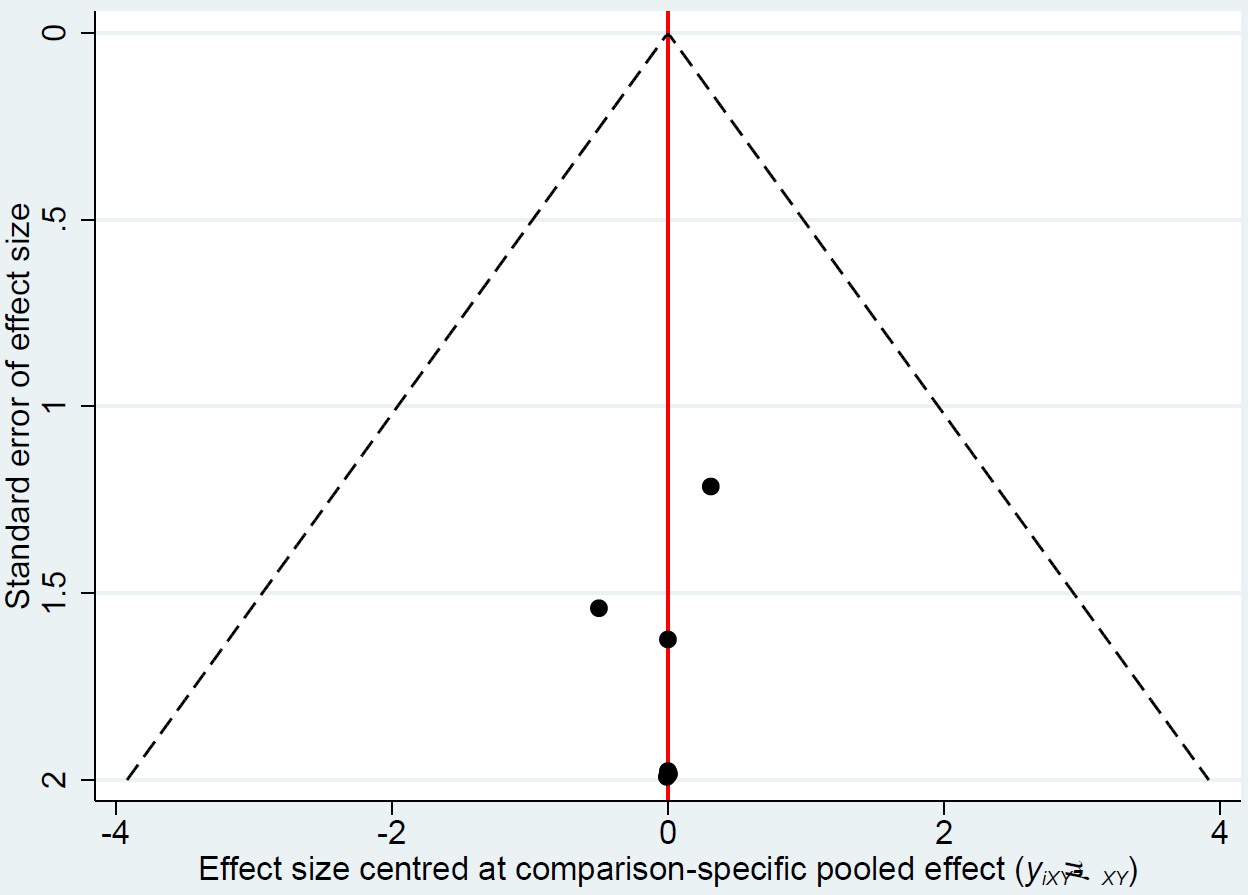


**Supplementary Figure 2** Funnel plot for transfuse red blood cell


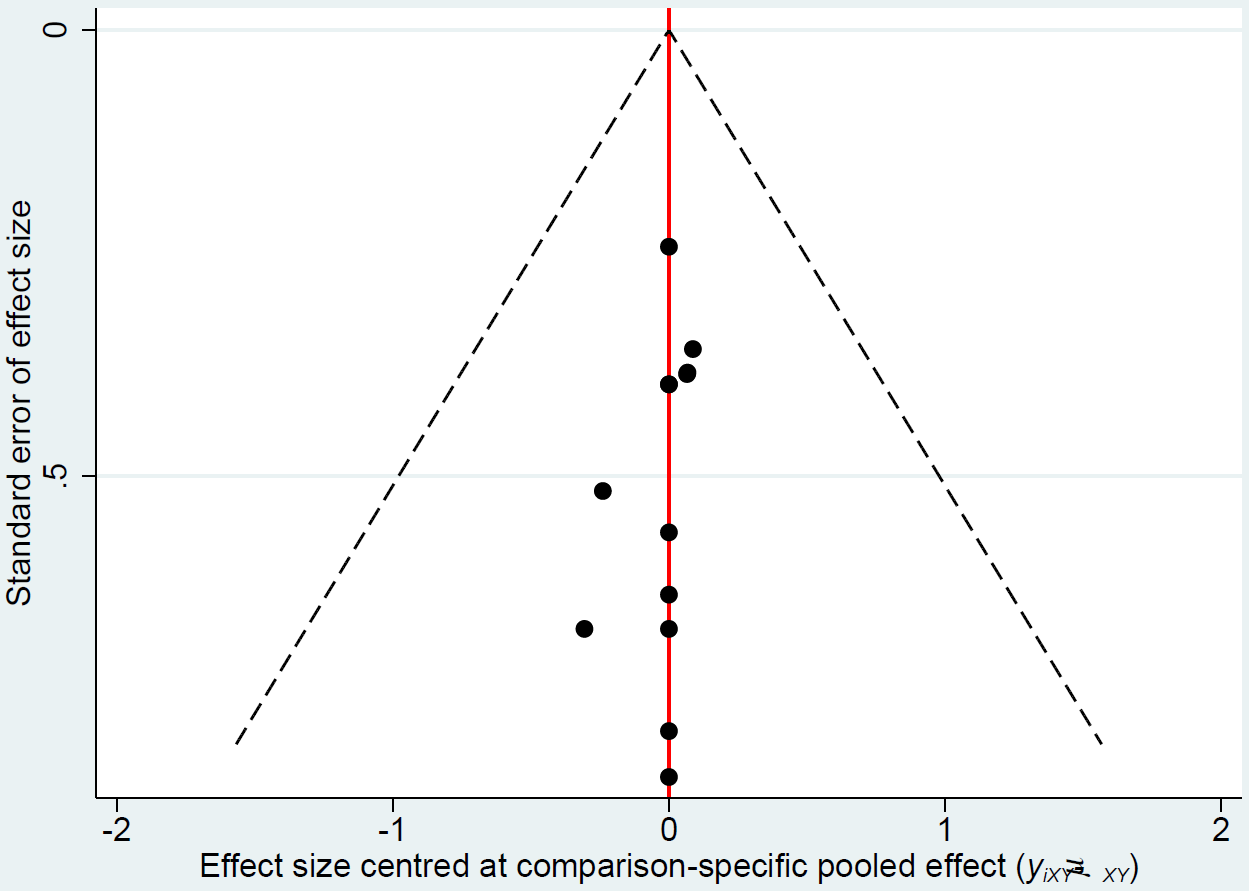


**Supplementary Figure 3** Funnel plot for transfuse fresh frozen plasma


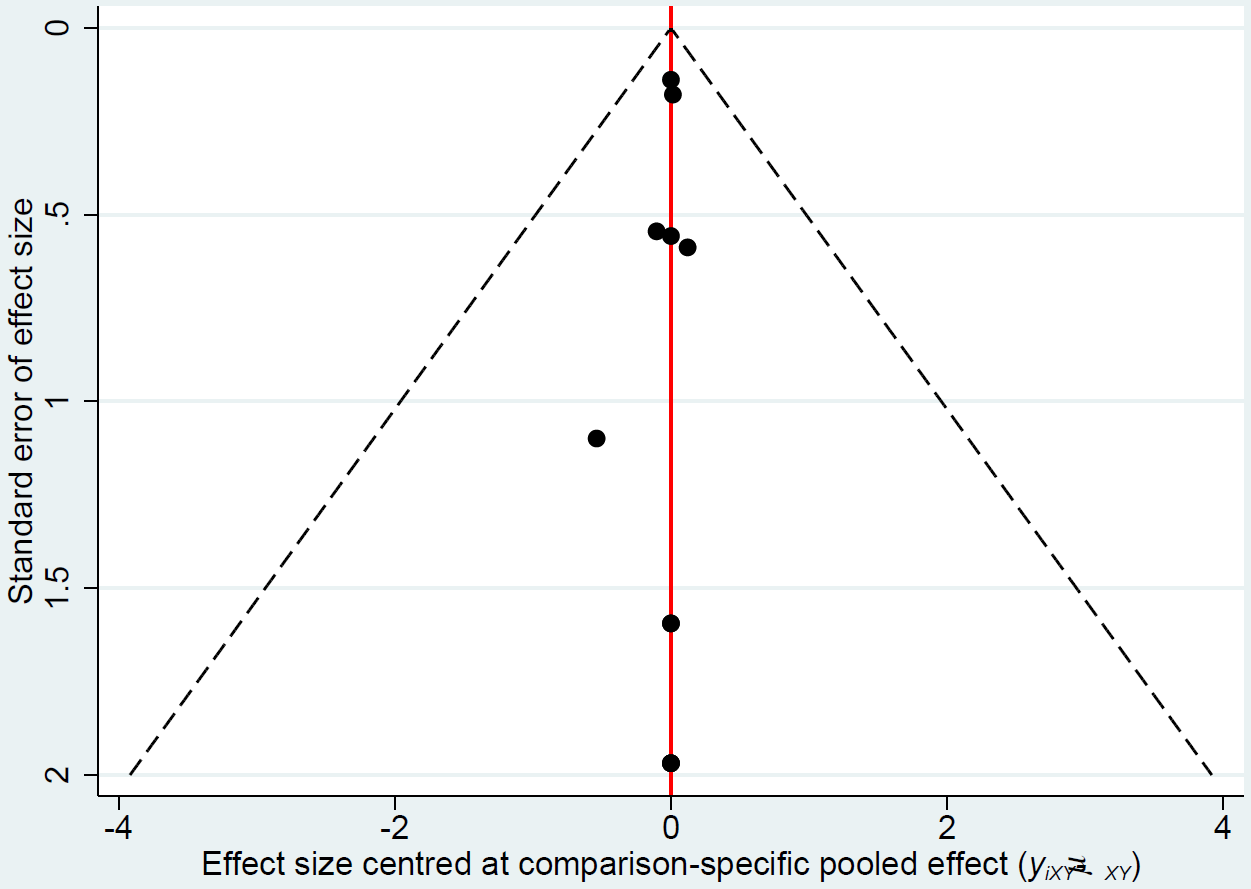


**Supplementary Figure 4** Funnel plot for transfuse platelet


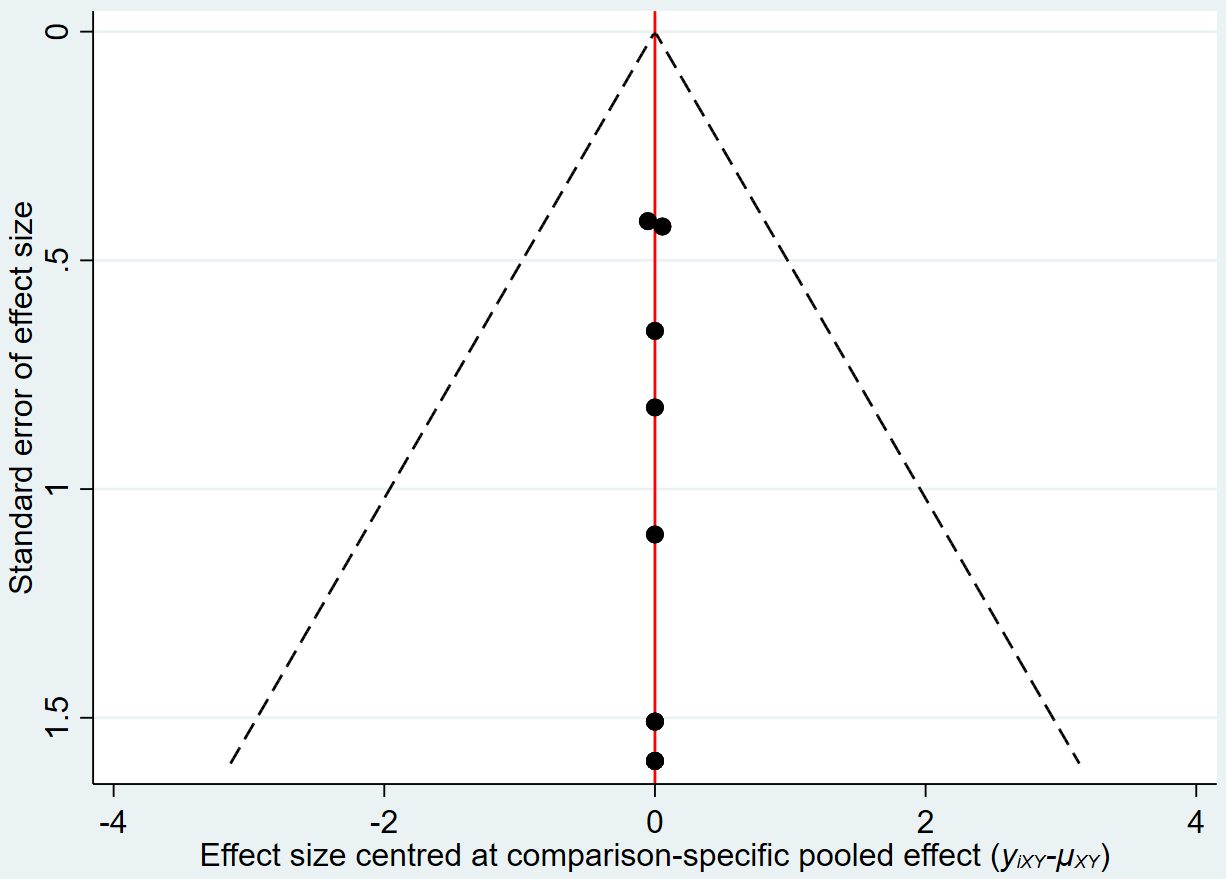


**Supplementary Figure 5** Funnel plot for urinary output at 24h after surgery


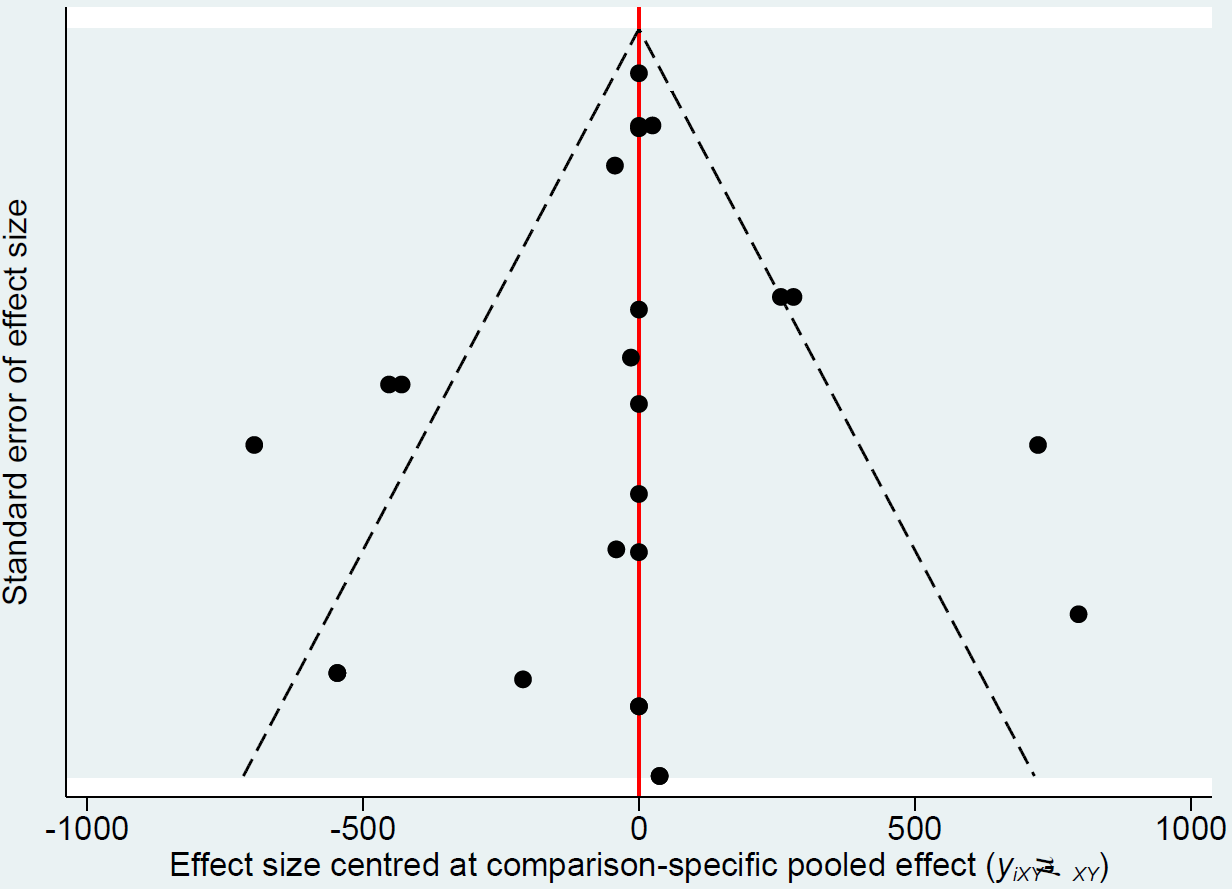


**Supplementary Figure 6** Funnel plot for postoperative chest tube output over the first 24h following surgery


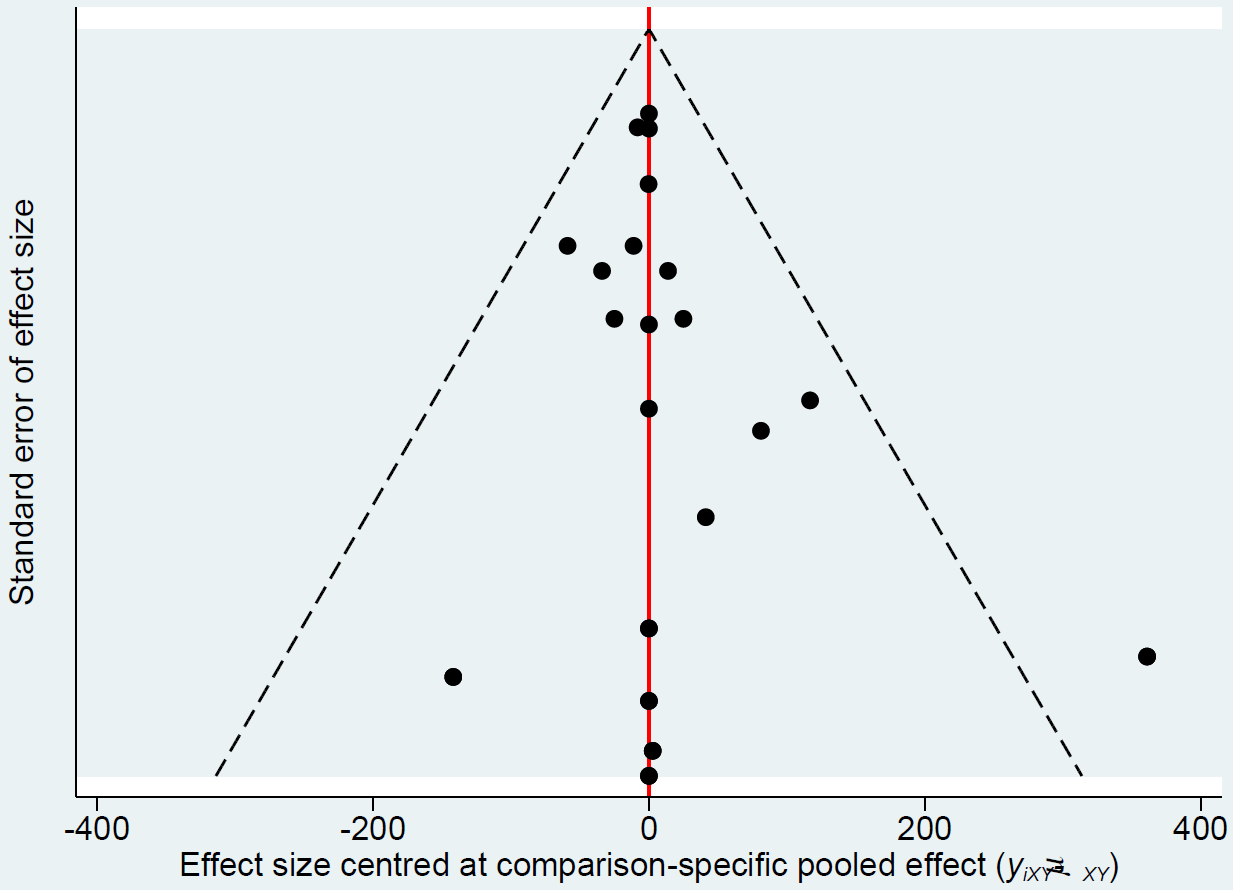


**Supplementary Figure 7** Funnel plot for length of ICU stay


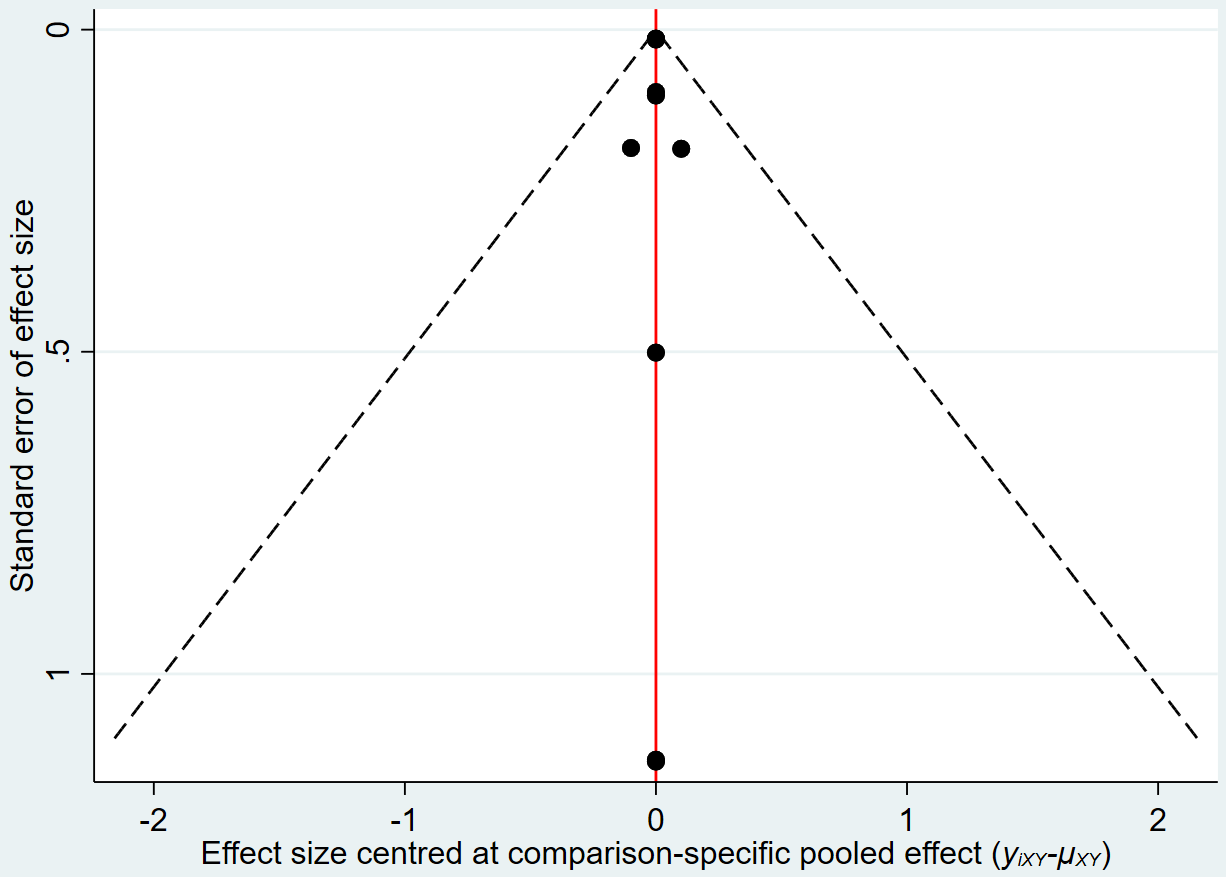


**Supplementary Figure 8** Funnel plot for length of hospital stay


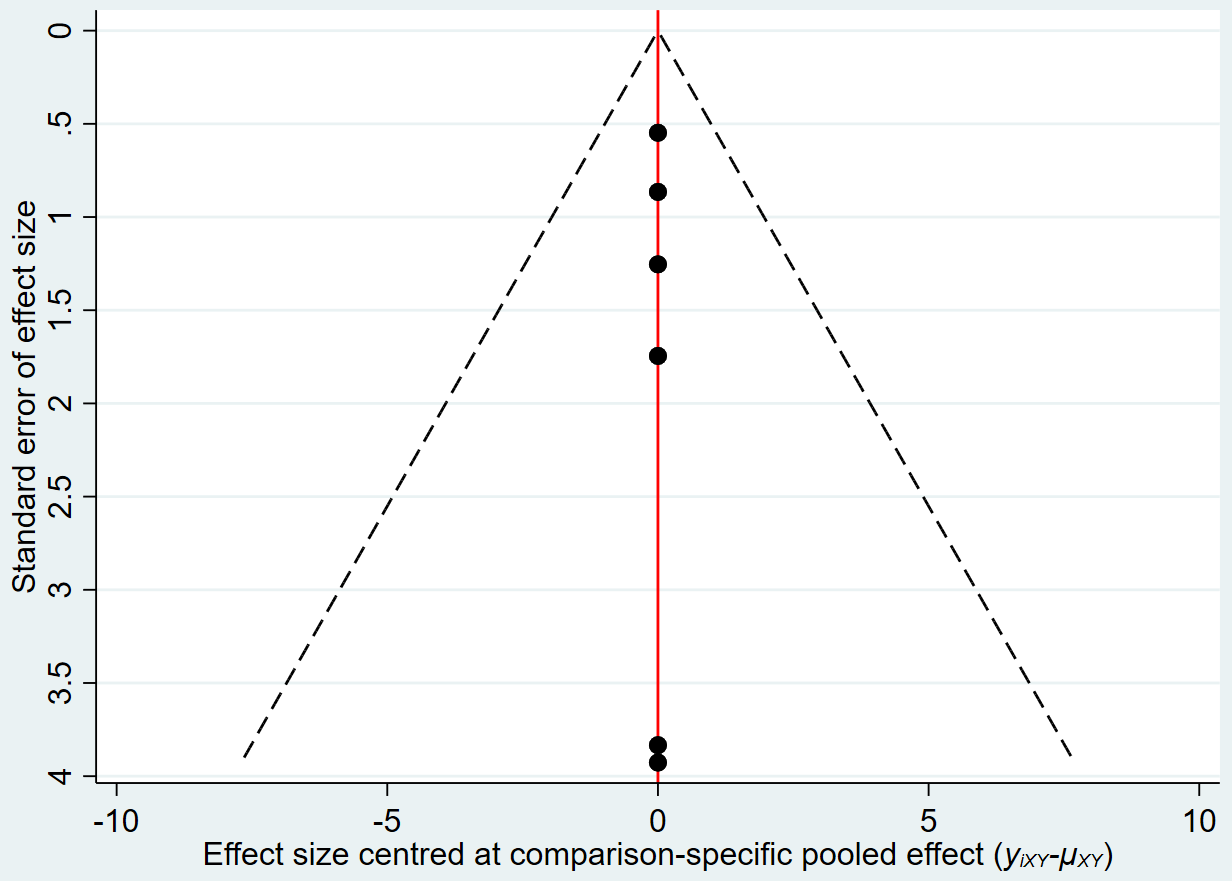

Supplement: Supplementary file 1 — Additional file1: Supplementary materials. [file 13741_2024_440_MOESM1_ESM.docx]
